# The effects of a prehabilitation programme based on therapeutic exercise, back care education, and pain neuroscience education in patients scheduled for lumbar radiculopathy surgery: A study protocol for a randomised controlled trial

**DOI:** 10.1371/journal.pone.0303979

**Published:** 2024-06-06

**Authors:** María Dolores Arguisuelas, Miriam Garrigós-Pedrón, Isabel Martínez-Hurtado, Juan Francisco Lisón, Gemma Biviá-Roig, Alejandro Álvarez-Llanas, Esteban Tortosa-Sipán, Rafael Llombart-Blanco, Víctor Rodrigo-Paradells, Matías Alfonso Olmos-García, Félix Tomé-Bermejo, Juan Francisco Blanco-Blanco, Julio Doménech-Fernández

**Affiliations:** 1 Department of Nursing and Physiotherapy, Universidad Cardenal Herrera CEU, CEU Universities, Valencia, Spain; 2 Department of Biomedical Sciences, Universidad Cardenal Herrera CEU, CEU Universities, Valencia, Spain; 3 Department of Orthopaedic Surgery, Hospital Arnau de Vilanova, Valencia, Spain; 4 Department of Orthopaedic Surgery, Clínica Universidad de Navarra, Pamplona, Spain; 5 Department of Orthopaedic Surgery, Hospital Universitario General Villalba, Madrid, Spain; 6 Department of Orthopaedic Surgery, Hospital Universitario Salamanca, Salamanca, Spain; University College Dublin - National University of Ireland: University College Dublin, IRELAND

## Abstract

The aim of this present clinical trial is to evaluate the effectiveness of a multicomponent prehabilitation programme administered through educational videos versus another programme based on written exercise recommendations, in patients scheduled for lumbar radiculopathy surgery. This study will be a multicentre, controlled, randomised, parallel clinical trial. One hundred participants undergoing lumbar radiculopathy surgery who meet the established inclusion criteria will be recruited at different Spanish hospitals. The experimental group will follow a 4-week prehabilitation programme combining therapeutic exercise, back care education, and pain neuroscience education delivered through videos designed for consumption at home. The control group will be provided with written instructions to perform therapeutic exercises during the same prehabilitation time period. The primary outcome of the study will be disability, assessed using the Spanish version of the Oswestry Disability Index. The secondary outcomes will be pain perception, health-related quality of life, fear avoidance, kinesiophobia, catastrophising, anxiety, depression, physical activity, and the treatment satisfaction of the patients. This study will provide evidence for the effectiveness of a home-based multicomponent prehabilitation programme that addresses some already identified barriers to patient attendance in face-to-face programmes. Understanding the medium and long-term effects of pre-surgery lumbar muscle training and pain neuroscience education administered via instructional videos watched by patients at home, will help improve the design of prehabilitation programmes in this population while also improving the cost-effectiveness of such interventions.

## Introduction

One of the most prevalent chronic pain conditions worldwide is low back pain (LBP), which affects more than 70% of the general population over their lifetimes and with an annual incidence of 40%. In addition, LBP tends to recur or become chronic, which can severely limit the daily quality of life of patients [1]. Lumbar radiculopathy is often cited as an indication for lumbar surgery, especially in the context of failed conservative care and worsening symptoms [2–4]. This condition is defined as unilateral leg pain worse than back pain, with pain radiating to the foot or toes and numbness and paresthesia in the same areas that is associated with a motor neurological deficit [5]. Surgical intervention for radiculopathy resulting from disc herniation and spinal stenosis has been found to yield substantial improvements at 3-month follow-up, yet only modest to moderate enhancements in terms of pain and disability are observed at 5-year follow-up assessments [6, 7]. Indeed, around 20% of patients undergoing surgery for lumbar radiculopathy develop chronic pain after surgery, which suggests that some patients may experience long-term pain and disability after lumbar surgery for radiculopathy, thereby leading to a high socioeconomic burden [8].

These patients usually require postoperative rehabilitation, even though the preoperative period is considered an ideal period for them to prepare for the surgery. Therefore, the concept of prehabilitation is characterized as the process of preparing patients to endure surgical stress more effectively, thereby facilitating expedited recovery through the enhancement of their functional capabilities and physiological reserves prior to undergoing surgery [9, 10]. In addition, prehabilitation has been demonstrated to decrease the likelihood of postoperative complications [11], thus aiding in the mitigation of postoperative pain intensity and duration of hospitalization, while also expediting patients’ return to preoperative levels of physical function[12].

In recent years, neuroscience education has emerged as a prominent educational strategy for patients with chronic LBP. Pain neuroscience education (PNE) is an education intervention based on cognitive learning that aims to reduce pain and disability by helping patients gain an increased understanding of their pain, de-emphasise the anatomopathological content, and focus on the factors that contribute to the development of pain, all within a biopsychosocial framework [13, 14]. PNE consists of educational sessions in which the physiology of the nervous system as well as the neurobiology of pain are explained to the patient in an easy to understand manner through the use of drawings, prepared pictures, examples, metaphors, and leaflets to supplement direct explanations [15]. Thus, PNE is based on improving patient knowledge about pain because, theoretically, it may reduce their pain. This is because pain is often related to fear and disability and so PNE can potentially help improve physical and mental health [16, 17].

Different studies have suggested that pre-surgery physiotherapy and pain education can improve outcomes, both in terms of function and health behaviour in patients with lumbar radiculopathy [11, 14]. Indeed, to date, 5 previous randomised clinical trials (RCTs) [11, 14, 18–20] have investigated the effect of different prehabilitation programmes in patients with lumbar disc herniation, degenerative lumbar spine disorders, or lumbar radiculopathy scheduled for lumbar surgery. First, Nielsen et al. [11] investigated the effects of a 2-month prehabilitation programme which included an intensive exercise programme and optimisation of the analgesic treatment. In turn, Louw et al. [14] investigated whether 1 session of PNE before surgery would improve the surgical outcome. Rolving et al. [18] examined whether a multidisciplinary cognitive-behavioural therapy intervention (6 sessions lasting 3 hours) would be beneficial. Next, in the study by Lindbäck et al. [19], patients received individual sessions of pre-surgery physical therapy twice a week for 9 weeks. Finally, Chu et al. [20] investigated the effects of virtual reality health education measures administered to the patients on the afternoon of the day they were admitted for scheduled surgery.

A recent meta-analysis including 5 RCTs found evidence with a high level of certainty supporting the usefulness of prehabilitation over standard care in terms of reducing preoperative back pain, as well as moderate-certainty evidence that prehabilitation improved health-related quality of life in patients that underwent lumbar surgery. Postoperatively, there was also moderate-certainty evidence supporting the notion that prehabilitation improved function following lumbar surgery after 6 months [21]. While on the one hand, there are currently no guidelines for rehabilitation planning before lumbar spine surgery, on the other, face-to-face prehabilitation may not be feasible for every patient. Therefore, some studies have highlighted the need for alternative preoperative lumbar spine education systems and so recommend exploring prehabilitation programmes based online [22].

To the best of our knowledge, to date, no studies have explored the possibility of a prehabilitation programme integrating patient physical conditioning through therapeutic exercise with PNE administered via videos watched by patients at home. We hypothesise that a multifactorial prehabilitation programme of this type, specifically designed for patients undergoing lumbar radiculopathy surgery, would yield well-informed and trained patients. We anticipate that this intervention would surpass the written instructions approach in terms of mitigating disability, improving quality of life, its the impact on psychological variables, and in the alleviation of back and leg pain. Therefore, the aim of this planned study will be to evaluate, in patients scheduled for lumbar radiculopathy surgery, the effects of a 4-week prehabilitation programme that combines therapeutic exercise, back care education, and PNE delivered through videos consumed by patients at home, compared to a regimen involving therapeutic exercise provided with written instructions. The outcomes we will study are disability, pain, quality of life, psychological variables, physical activity, and the satisfaction of patients with the treatment.

## Materials and methods

### Study design

This study will be a multicentre, controlled, randomised, parallel clinical trial. This study protocol was written in accordance with the Standard Protocol Items: Recommendations for Interventional Trials (SPIRIT) guidelines [23], with the aim of improving the quality of the eventual clinical trial.

### Ethical approval and registration

The design of this study conforms to the principles outlined in the Declaration of Helsinki and the protocol was approved by the Research Ethics Committee at University CEU Cardenal Herrera (reference number CEEI23/459). Participation in the study will be voluntary and will require the written informed consent of each participant. Eligible patients will be informed about all the relevant aspects of this study before starting their rehabilitation programme. The protocol was registered with the United States National Library of Medicine (ClinicalTrials.gov) with identifier NCT06145620 in November 2023. Personal information will be collected by the clinical research coordinators and stored in a database on a password-protected computer in order to protect patient confidentiality.

### Participants: Recruitment and eligibility criteria

One hundred patients will participate in this study and their recruitment is expected to start in January 2024 and continue up to January 2025. The inclusion criteria will be the following: adults aged 18 to 80 years diagnosed with lumbar radiculopathy to whom surgery has been proposed. The predominant symptoms that will justify treatment by surgical decompression will be leg pain, with or without neurological deficits. Participants will be excluded if: (1) they are undergoing any other non-pharmacological treatments or physical therapy designed to address lumbar radiculopathy; (2) surgery with instrumentation (e.g., spinal fusion, arthrodesis, etc.) has been proposed to them; (3) they require acute surgery; (4) they have a concurrent chronic pain condition (e.g., fibromyalgia, chronic fatigue syndrome, etc.); (5) they exhibit symptoms of spinal cord compression; (6) they have been diagnosed with a malignant tumour; (7) they have a mental illness; (8) they have undergone a previous spinal surgery; or (9) they do not have access to an internet-enabled mobile device.

### Randomisation and blinding

The sample will be recruited by the physicians in the Orthopaedic Surgery Services at different Spanish hospitals, who will also be responsible for obtaining the signed informed consent from the enrolled patients. A statistician from outside this research team will generate the random sequence using random number allocation software [24]. Participants will be randomly assigned to the experimental group (EG) or the control group (CG). To achieve a balanced distribution within each group according to sex and age, these variables will be blocked obscured during the patient assignment process. Additionally, randomisation will occur in blocks of 2 participants to ensure equality in the number of patients assigned to each group and also facilitating the possibility of partial and/or interim analyses.

The randomisation sequence will remain concealed throughout the application of the intervention programme and entire data collection procedure. Only the investigator responsible for patient group assignment will have knowledge of this sequence. Thus, the remaining researchers responsible for collecting all the pre-intervention (baseline), post-intervention (immediately after the 4-week prehabilitation), and follow-up assessment (1 month, 6 months, and 12 months after surgery) data will be blinded to the participant group assignments. It will be impossible to blind the participants to their group assignments because of their active role in performing the interventions and the evident differences between their intervention types.

### Intervention

Participants enrolled in the EG will undergo a 4-week prehabilitation programme structured around 3 components: therapeutic exercise, education about back and spine care, and PNE. These interventions will be self-administered by the patients while at home, facilitated by watching a series of educational videos provided by us.

1. Therapeutic exercise

The participants will engage in targeted therapeutic exercises designed to enhance their strength and neuromuscular control of the abdominal and spinal erector muscles. To this end, we will meticulously produce and edit a series of instructional videos to showcase the correct execution of these exercises. These therapeutic exercise videos will span a wide range of difficulty levels to accommodate the varying physical capacities of the individual participants. The baseline scoring obtained from the International Physical Activity Questionnaire-Short Form (IPAQ-SF) will be used to ascertain the most appropriate exercise level for each participant. Thus, videos appropriately tailored to the level of each participant will be distributed within the first week of the prehabilitation programme. During the 4 weeks leading up to the scheduled surgical intervention, participants will undertake 5 weekly sessions of therapeutic exercise at home, guided by the detailed explanations provided in the videos. Furthermore, patients will also undergo mid-programme monitoring by a designated investigator who will establish contact with them to motivate compliance with the exercise regimen and address any queries or concerns the participants may have.

2. Spinal care education

Patients will receive education on spinal care by watching an informative video on the subject. The aim of this material will be to provide information to the patients about the proper execution of movements with their spine and methods for protecting their back. Self-care specifically for the spine will be addressed, including examples of the proper postures to use during the daily activities of life (e.g., walking, standing, sitting, lying down, getting up, sleeping, lifting heavy objects, performing household tasks, and occupational postures, etc.). This material will be distributed within the first week of the prehabilitation programme.

3. Pain neuroscience education

Finally, participants will also be taught the neuroscience of pain by watching educational videos on the topic. The aim of this material will be to help the patients reconceptualise their pain by shifting attention away from the nociception of the affected areas, instead focusing on perceiving pain as an increase in nerve sensitivity and the ascending regulation of the peripheral and central nervous systems. The goal of the PNE will be to reduce patient anxiety and uncertainty and to foster positive expectations and beliefs regarding their scheduled surgeries. The topics covered in the different videos will include education on the physiology of pain, decision-making about undergoing surgery, the goals of surgery, and postoperative recovery [8].

We will produce and edit 3 videos covering the aforementioned monographic content, each lasting approximately 15 minutes. These videos will be distributed sequentially during the last week of the prehabilitation programme, following the recommendations for the optimal timing of PNE in preoperative patients [13, 25]. Patients will be instructed to watch these videos promptly after their distribution and to recall the content provided to them throughout the whole programme. Participants allocated to the CG will be provided with written instructions, by mail, detailing how to perform the back strengthening exercises and will be advised to adhere to the same regimen as the EG during the 4-week prehabilitation programme. In both patient groups, compliance with the exercises will be assessed by daily self-reporting in a logbook. Finally, the anaesthesia and surgical procedures used for the patients in both groups will be similar.

### Outcomes and measurements

Sociodemographic data (including participant sex, age, height, weight, BMI, smoking status, educational level, employment situation, pain duration and medication intake) will be collected prior to the implementation of the prehabilitation programme. Additionally, information will also be gathered on the education level, employment status, duration of symptoms, medication use, and level of physical activity the patients engage in.

### Primary outcome

The primary outcome, the degree of disability, will be measured using the Spanish version of the Oswestry Disability Index (ODI) [26] which has been validated for measuring disability in the Spanish population with LBP and in Spanish patients that have previously undergone lumbar disc surgery [27]. The ODI is widely used in the fields of neurosurgery and spine surgery [28] and is considered the gold standard test for measuring disability. The ODI comprises a total of 10 items that ask about pain intensity, personal care (bathing, dressing, and so on), lifting, walking, sitting, standing, sleeping, sex life, social life, and travelling. Each item has 6 grades, and the higher the score, the more serious the disability. A difference of 15 points has been suggested as the minimum clinically important difference (MCID) for surgical populations [29].

### Secondary outcomes

Pain perception will be assessed using the Spanish version of the Short Form McGill Pain Questionnaire (SF-MPQ) [30] and a numerical rating scale (NRS). The SF-MPQ consists of a 15-point descriptor of average pain, articulated as 11 points of sensory experience and 4 points of affective experience. Together, the sensory and affective pain rating scores give a value for a total pain experience ranging from 0 (no pain) to 45 (maximum pain). Melzack [31] previously showed that the SF-MPQ is a responsive scale that provides both reliable and valid data. The suggested MCID for the SF-MPQ is 5 points [32]. An 11-point (0–10) NRS will also be employed to measure pain intensity, as previously recommended in clinical trials of chronic pain treatments [33]. The NRS will present the numbers 0 to 10 to the patients, where 0 means ‘no pain’ and 10 means ‘the worst pain imaginable’. The participants will be asked to rate their pain by indicating the number that best describes their average pain over the 24 hours prior. The suggested MCID for this variable is 2 points for patients with chronic pain [34].

Health-related quality of life will be measured using the Spanish version of EuroQol-5D (EQ-5D) [35]. The EQ-5D is a self-reported questionnaire comprising 5 dimensions: mobility, self-care, usual activities, pain/discomfort, and anxiety/depression, with each dimension subdivided into 3 levels: ‘no problems’, ‘some problems’, and ‘extreme problems’, indicating the level of function perceived by the patient. Each level carries a weighted score which is combined across the 5 dimensions to reach an overall index score. EQ-5D scores range from −0.5 to 1.0, with negative scores indicating states ‘worse than death’, 0 indicating no quality of life or ‘death’, and 1 indicating full health. The EQ-5D has previously been used as an outcomes indicator in patients with lumbar pain [19, 36].

The Spanish version of the Fear-Avoidance Beliefs Questionnaire (FABQ) [37] is a self-reported questionnaire consisting of 16 independent sentences rated by the participant on a 7-point Likert scale ranging from 0 (‘completely disagree’) to 6 (‘completely agree’), with higher scores reflecting higher levels of fear-avoidance beliefs. The questionnaire contains 2 subscales: the FABQ-Work (ranging from 0 to 42 points) and FABQ-Physical Activity (ranging from 0 to 24 points), which assess the patient’s attitudes and beliefs about how occupational or physical activities may influence their LBP, respectively. The suggested MCID value of the FABQ-Physical Activity subscale is 6 points [38]. Moreover, the FABQ has been shown to have good reliability and validity [39].

Kinesiophobia will be measured with the Spanish version of Tampa Scale for Kinesiophobia (TSK-11). This is a self-reported scale in which patients rate 11 statements about their fear of movement on a 4-point Likert scale. This results in a score range of 11 to 44 points, with higher scores corresponding to a greater fear of pain, movement, and injury. The TSK-11 has also been shown to have good reliability and validity [40].

The level of pain catastrophising will be assessed using the Spanish version of Pain Catastrophizing scale (PCS). The PCS measures 3 related constructs of pain catastrophising: magnification, rumination, and helplessness, with a total score ranging from 0 to 52 points. Participants will be asked to score 13 pain-related cognitions on a 5-point Likert scale, where higher scores indicate a higher degree of pain catastrophising. The internal consistency of the Spanish version of the PCS is high (α = 0.94) [41].

Anxiety and depression will be measured with the Spanish version of the Hospital Anxiety and Depression Scale (HADS) [42]. The HADS comprises two 7-item scales designed to rate depression (HADS-D) and anxiety (HADS-A). Each item is scored from 0 to 3, with a score range on each subscale of 0 to 21 points. Scores of 0–7 indicate the absence of anxiety or depression; 8–10 indicate mild levels; 11–14 indicate moderate levels; and 15–21 indicate severe levels of anxiety or depression. The Spanish version has shown suitable levels of reliability (for HADS-D α = 0.82 and for HADS-A α = 0.81).

Physical activity levels will be measured using the IPAQ-SF validated for Spanish. This is a self-administered questionnaire comprising 7 items which collects information about the physical activity the surveyee has engaged in during the 7 days prior. This questionnaire collects information about the days per week and minutes per day the respondents spent engaged in vigorous or moderate exercise, walking, and sedentary activities. The validity of the IPAQ-SF has been shown to be acceptable for the measurement of total and vigorous physical activity and its reliability coefficients for application in the Spanish population were good [43].

Patient satisfaction with the treatment will be assessed using the Patient Global Impression of Change (PGIC) scale to allow them to express their overall impression of how they have changed following the prehabilitation programme. The PGIC is measured on a 7-point Likert scale where 1 represents ‘completely recovered’, 2 is ‘very much improved’, 3 is ‘slightly improved’, 4 corresponds to ‘no change’, 5 is ‘a little worse’, 6 is ‘much worse’, and 7 is ‘enormously worse’. Improvement will be considered with a score of 1, 2, or 3; the absence of change, a score of 4; and scores of 5, 6, or 7 will be considered patient deterioration. Of note, this scale has been consistently employed to evaluate patient satisfaction in cases of chronic lumbar pain [19, 44].

All the primary and secondary variables will be assessed using a form distributed to the participants by email at different points throughout the study (baseline, immediately after the 4-week prehabilitation, and at the 1-month, 6-month, and 12-month post-surgery follow-up assessments). After completion of the prehabilitation programme, it will be verified that participants have not interacted with other participants through a question specifically designed for this purpose in the form. Similarly, participants will also be asked about the number of videos viewed, and their level of assimilation and comprehension will be assessed through a brief quiz on the material’s content.

**Fig 1:**
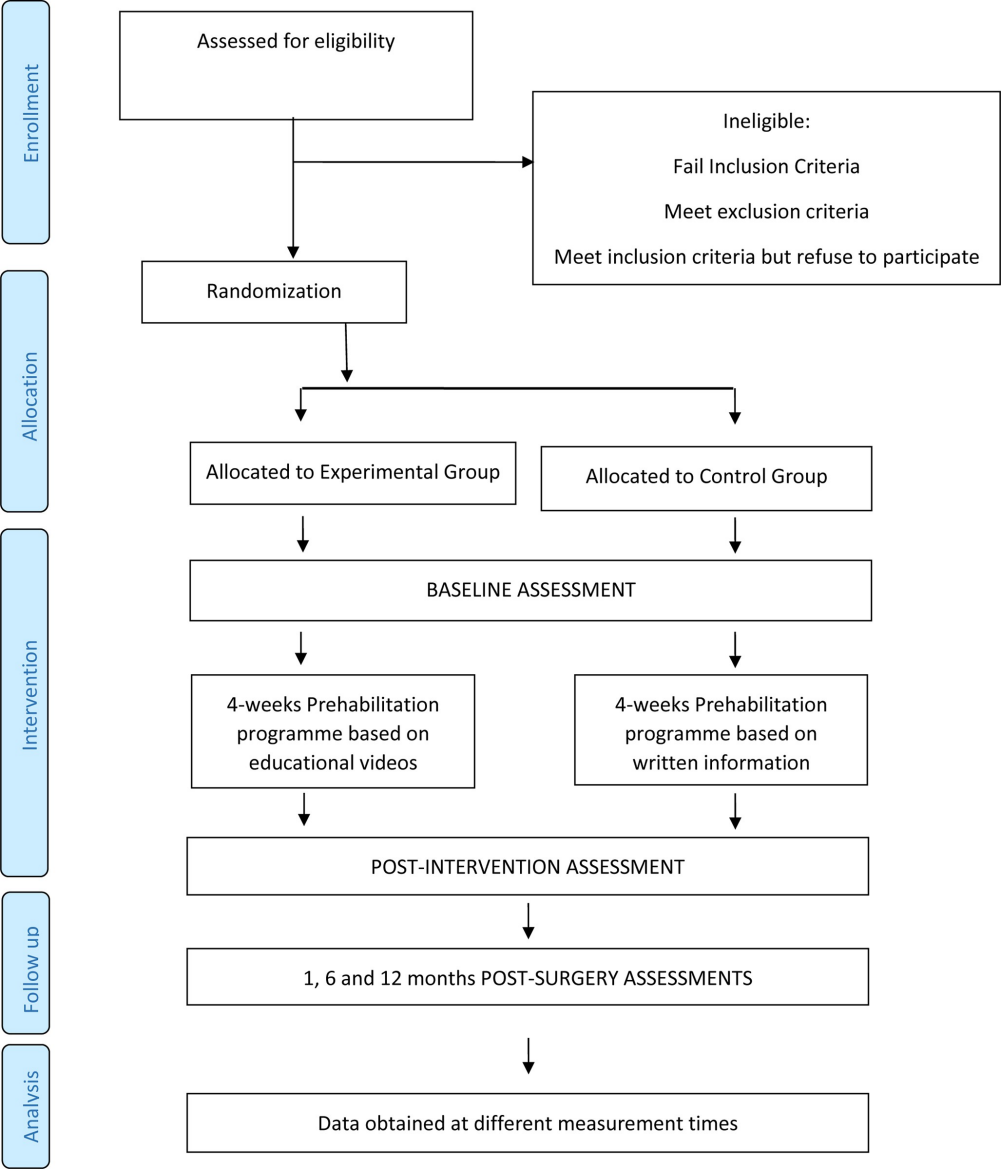
Protocol timeline implemented for the study.

All the adverse events will be evaluated, recorded, and discussed in the final communication of the study results. The study design and progression of patients through the study protocol are outlined in Fig 1.

### Statistical methods

The sample size has been determined a priori using G-Power software (version 3.1.9.2)(F tests, ANOVA: Repeated measures, within-between interaction). This calculation was based on the results of a similar study evaluating the effects of a prehabilitation programme in patients with degenerative lumbar spine disorders [19]. In this previous study, an effect size (Cohen *d*) of 0.3 was observed in the primary variable (measured using the ODI) after completion of the preoperative treatment. Therefore, considering an alpha probability of 0.05, an observed power of 0.9, and the effect size found by Lindbäck et al. (*d* = 0.3), we determined that the inclusion of a total of 82 participants will be required. However, to account for potential losses, we will increase the sample size by 20%, meaning that a total of 100 patients will be included in this work, 50 in each group.

The statistical analysis will be conducted by taking an intention-to-treat approach. Missing data will be handled using the multiple imputation method, on the assumption that values at each time point follow a specific distribution. For between-group comparisons of demographic data, unpaired Student *t*-tests or Mann–Whitney U tests will be used for continuous variables and chi-squared tests will be employed for categorical variables. Two-way analysis of covariance and repeated measures (with age and sex as covariates) will be used to compare the effects of the interventions in each group over time. Analysis of covariance will be adjusted for baseline values. A confidence interval of 95% will be used to establish differences and statistical significance will be reported for all between-group differences at *ρ* < 0.05. Cohen *d* effect sizes will also be calculated for changes in groups over time, where a Cohen *d* of 0.20 will be considered a small, 0.50 a medium, and 0.80 to infinity a large effect size, respectively [45]. All the statistical analyses will be performed using SPSS software for Windows (version 27.0, IBM. Corp., Armonk, NY).

### Data management

All the data will be entered into a database using unique study codes for each participant and will be stored securely on password-protected computer. Only the data manager, who is independent from competing interests, will be able to access to the data. A Data Monitoring Committee will not be required because the study will carry minimal risk. Any important protocol modifications made during the study period will be communicated to the trial registry and the journal in which this protocol is published.

## Discussion

To the best of our knowledge, this will be the first randomised clinical trial to explore the efficacy of a prehabilitation programme combining therapeutic exercise, back care education, and PNE in patients scheduled for lumbar radiculopathy surgery and delivered by having the patients watch instructional videos at home. There is consensus that the preoperative phase is an opportune period for patient preparation preceding surgery. However, the prehabilitation programmes designed for patients undergoing surgery for lumbar radiculopathy published to date have predominantly emphasised either the implementation of therapeutic exercise alone or the use of different one-on-one or group educational session methods. Thus, various previous studies have developed different prehabilitation programmes based on exercise [11], cognitive behavioural therapy [18], PNE [14], physiotherapy combined with a behavioural approach [19], or virtual reality health education [20] in patients with lumbar disk herniation, degenerative lumbar spine disorders, or lumbar radiculopathy, scheduled for lumbar surgery.

However, some factors such as travel distance, transportation links, parking difficulty, and cost have been recently identified as barriers to patient attendance of such interventions [46]. Therefore, developing prehabilitation programmes that address these factors could facilitate the expansion of engagement with prehabilitation to a larger number of patients. In this regard, this present study protocol proposes the implementation of a home-based multicomponent prehabilitation programme which also represents a cost-effective means of intervention. Nevertheless, we must acknowledge as a limitation that the time commitment in viewing videos and reading may differ between the two groups, as we will compare a multimodal intervention delivered through videos versus the treatment as usual.

This study will provide evidence about both the short-term and long-term outcomes of pre-surgery lumbar muscle training and PNE by having patients watch educational videos at home. Consequently, the results of this study will help improve the design of prehabilitation programmes specifically tailored to patients with lumbar radiculopathy that are scheduled for surgery.

## Supporting information

S1 Data Trial protocol study_Spanish version. (DOCX)

S2 Data Trial protocol study_English version. (DOCX)

S3 Data PLOS one human subjects research checklist. (DOCX)

S1 File SPIRIT checklist. (DOC)
