# Supplementary material for: The effects of a prehabilitation programme based on therapeutic exercise, back care education, and pain neuroscience education in patients scheduled for lumbar radiculopathy surgery: A study protocol for a randomised controlled trial
Source: PLoS One. 2024 Jun 6;19(6):e0303979. doi: 10.1371/journal.pone.0303979 (PMC11156268; doi:10.1371/journal.pone.0303979)
Supplement: S1 Data — (DOCX) [file pone.0303979.s001.docx]

Memoria solicitud comité de ética para la investigación biomédica

Título: EFICACIA DE UN PROGRAMA DE PREHABILITACIÓN BASADO EN LA REALIZACIÓN DE EJERCICIO TERAPÉUTICO, EDUCACIÓN SOBRE CUIDADOS DE LA ESPALDA Y EDUCACIÓN EN NEUROCIENCIA DEL DOLOR, EN PACIENTES QUE VAN A SER INTERVENIDOS POR RADICULOPATÍA LUMBAR

Nombre de IP proyecto de investigación: María Dolores Arguisuelas Martínez

**TÍTULO:** EFICACIA DE UN PROGRAMA DE PREHABILITACIÓN BASADO EN LA REALIZACIÓN DE EJERCICIO TERAPÉUTICO, EDUCACIÓN SOBRE CUIDADOS DE LA ESPALDA Y EDUCACIÓN EN NEUROCIENCIA DEL DOLOR, EN PACIENTES QUE VAN A SER INTERVENIDOS POR RADICULOPATÍA LUMBAR

**RESUMEN:**

Actualmente, el abordaje terapéutico de los pacientes que se intervienen por una radiculopatía lumbar se basa en la rehabilitación postquirúrgica, sin embargo, son escasos los estudios que analizan los efectos de programas de prehabilitación, es decir, programas dirigidos a mejorar la preparación y capacidad funcional del paciente antes de la cirugía. El presente proyecto se propone la evaluación de un programa de prehabilitación, de aplicación durante las 4 semanas previas a la intervención quirúrgica, basado en tres ejes terapéuticos complementarios entre sí: realización de ejercicio terapéutico, educación sobre los cuidados de la columna vertebral y educación sobre neurociencia del dolor. Se evaluarán los efectos producidos sobre variables de la condición de salud del paciente (dolor, discapacidad y calidad de vida) y variables comportamentales (creencias de temor evitación, catastrofismo, ansiedad y depresión). En conclusión, se propone el desarrollo de un programa de prehabilitación dirigido a pacientes con dolor lumbar, generando un programa coste-efectivo, que pueda ser auto-aplicado en su mayor parte, y que aumente y mantenga la motivación para promover una mejor preparación (tanto física como emocional) de cara a la cirugía, pudiendo desembocar todo ello en una mejor recuperación postquirúrgica y, por tanto, una menor utilización de los servicios sanitarios.

**INTRODUCCIÓN Y ANTECEDENTES:**

Relevancia del dolor lumbar como un problema de salud pública: impacto sobre la discapacidad y la calidad de vida

El dolor lumbar es una de las afecciones de dolor crónico más prevalentes en todo el mundo, que afecta a más del 70% de la población en general a lo largo de la vida, con una incidencia anual del 40%. Además, muestra una gran tendencia a cronificarse o producir recurrencias que pueden limitar gravemente la vida diaria de los pacientes (Balague et al., 2012). En una encuesta realizada en la población española, la prevalencia a un año fue del 20% para el dolor lumbar (Fernández de las Peñas, 2011). Actualmente es el motivo más frecuente de baja laboral y la principal causa de discapacidad en las persones menores de 45 años. El coste económico atribuible a LBP es de 8945,6 millones de euros anuales, representando el 0,68% global del Producto Interior Bruto español (Alonso-García 2020). En nuestro país, el dolor lumbar es la primera causa de discapacidad ajustada por años de vida, superando la enfermedad cardíaca (Soriano, 2018).

La sintomatología y la función de la columna se pueden mejorar mediante la rehabilitación en la mayoría de los pacientes, sin embargo, del 10% al 20% de los pacientes requieren cirugía (Liu et al., 2019). El período postoperatorio agudo está asociado con una marcada reducción en la función física y la calidad de vida relacionada con la salud (Carli y Mayo 2001). La hospitalización postoperatoria suele durar de 1 a 3 días y es común comenzar la rehabilitación física de 4 a 6 semanas después de la cirugía (Oosterhuis et al., 2017). Aproximadamente el 80% de los pacientes regresan al trabajo 12 meses después de la cirugía (Nygaard et al., 1994). Las razones son variadas e incluyen la selección de pacientes, los aspectos psicosociales y la variabilidad en el espectro de la enfermedad degenerativa del disco, aunque la rehabilitación postoperatoria también tiene una influencia importante en los resultados quirúrgicos (Choi et al. 2005). Sin embargo, recientemente, Paulsen et al. observaron que la derivación del paciente a un grupo de rehabilitación individualizada supervisada o a un grupo domiciliario sin planificación de ejercicios, no afecta a la duración de la baja por enfermedad postoperatoria, el regreso al trabajo o la capacidad laboral en pacientes que se recuperan después de una cirugía por hernia de disco lumbar. Por contra, sí encontraron asociación significativamente entre la duración del dolor irradiado de las piernas y la capacidad de trabajo durante la fase preoperatoria con la duración de la baja por enfermedad (Paulsen et al., 2020).

La hernia discal lumbar es una causa común de dolor y discapacidad en la población general (Gadjradj et al., 2017). De hecho, en pacientes con radiculopatía lumbar, se ha estimado que un porcentaje entre el 10% y el 40% puede experimentar dolor y discapacidad persistentes incluso después de la discectomía (Lurie et al., 2003; Weinstein et al., 2006). La discapacidad y la calidad de vida están interrelacionadas y se afectan mutuamente. Por lo tanto, controlar la discapacidad puede promover la salud, mejorando así la calidad de vida (Chu et al., 2020). Del mismo modo, independientemente de la etiología, la estenosis lumbar puede causar dolor crónico y discapacidad, reduciendo dramáticamente la calidad de vida, movilidad y función (Chad 2007).

La tasa de éxito de la cirugía en las intervenciones por radiculopatía lumbar se estima entre el 75% y el 80% (Atlas et al., 2001, Asch et al., 2002). A pesar de la alta tasa de éxito quirúrgico, del 23% al 28% de los pacientes sometidos a una cirugía de descompresión terminan con dolor crónico de espalda o de piernas (Atlas et al., 2001, Voorhies et al., 2007, Kreiner et al., 2014). Estos resultados desfavorables están asociados con niveles más altos de utilización de la atención médica postoperatoria, incurriendo en mayores costos de atención de la salud (Taylor y Taylor 2012) y dando lugar a una alta carga socioeconómica.

El desarrollo de dolor crónico postquirúrgico puede deberse a una multitud de factores, incluidos, entre otros, factores psicológicos negativos, dolor crónico preexistente, trastornos psicológicos, alta sensibilidad de la proteína C reactiva o dolor preoperatorio articular (Voorhies et al., 2007, Klinger et al., 2008, Rathod et al., 2014). Así pues, parece que la experiencia de dolor del paciente además de los atributos del individuo hacia el dolor juegan un papel muy importante en la recuperación de esta afección. Por este motivo, uno de los ejes principales de la intervención que se propone en el presente Proyecto se basa en la educación al paciente sobre neurociencia del dolor, con el objetivo de reducir el miedo asociado con el dolor lumbar, al proporcionar información al paciente sobre la neurofisiología, procesamiento y representación de la experiencia de dolor.

Por otro lado, teniendo en cuenta que la rehabilitación postoperatoria ha mostrado un efecto limitado en la reducción del dolor y la discapacidad, es necesario considerar otras estrategias diferentes de las que se han desarrollado hasta el momento, que puedan incidir sobre dichas condiciones de salud. Es por ello que el presente Proyecto propone una intervención preoperatoria dirigida a mejorar la capacidad funcional del paciente antes de la cirugía y sus estrategias de afrontamiento ante la misma.

Neurociencia del dolor

En las últimas décadas, el modelo biopsicosocial se ha aplicado como marco para comprender la complejidad del dolor crónico en general, y el dolor lumbar en particular, preferentemente a un enfoque puramente biomédico. Desde esta perspectiva, muchos factores, incluidos los biofísicos, psicológicos, sociales y genéticos, y las comorbilidades pueden contribuir a la discapacidad en el dolor lumbar.

No obstante, actualmente, en el contexto del paciente con dolor lumbar que debe enfrentarse a una cirugía, el asesoramiento preoperatorio que recibe el paciente se centra, principalmente, en modelos biomecánicos y anatómicos para el abordaje del dolor y la discapacidad (Brox et al. 2008, Moseley 2004). Este tipo de información, no solo ha demostrado una eficacia limitada, sino que incluso, puede aumentar los miedos, la ansiedad y el estrés en pacientes con radiculopatía lumbar que van a recibir una intervención quirúrgica (Maier-Riehle et Harter 2001, Louw et al., 2014).

Recientes investigaciones sobre estrategias educativas para pacientes con dolor lumbar muestran un incremento en el uso de la educación en neurociencias terapéuticas. Ello tiene como objetivo reducir el dolor y la discapacidad ayudando a los pacientes a adquirir una mayor comprensión de los aspectos biológicos y procesos fisiológicos implicados en su experiencia de dolor. La neurociencia del dolor es una intervención de base cognitiva que se diferencia de las estrategias de educación tradicionales en que no se centra en aspectos anatómicos o biomecánicos, sino más bien sobre neurofisiología, neurobiología, procesamiento y representación, y significado del dolor. En resumen, la neurociencia del dolor resta importancia a los modelos de dolor tradicionales basados en tejidos anatómicos (Moseley 2003a, Melzack 2001), y tiene como objetivo reducir el miedo asociado con el dolor lumbar al proporcionar más información sobre el dolor y la neurofisiología de una experiencia de dolor.

Una revisión sistemática, llevada a cabo por Louw y cols. (2011), sugiere evidencia sólida de la educación con neurociencia del dolor sobre el dolor, discapacidad y rendimiento físico en dolor musculoesquelético, particularmente en trastornos de la columna. Concretamente, los efectos observados fueron una disminución del miedo y cambios en la percepción del dolor (Moseley 2003b); un efecto inmediato sobre las actitudes ante el dolor (Moseley 2003a); mejoras en el dolor, cognición y rendimiento físico (Moseley 2004a); aumento de los umbrales del dolor durante las tareas físicas (Moseley et al., 2004b); mejores resultados en la realización de ejercicios terapéuticos (Moseley 2002); y reducción significativa de la actividad cerebral característica en la experiencia de dolor (Moseley 2005).

Por otro lado, una revisión Cochrane realizada en pacientes con dolor lumbar crónico concluyó que, probablemente, los pacientes que reciben una rehabilitación biopsicosocial multidisciplinaria experimentan menos dolor y discapacidad que los que reciben la atención habitual o un tratamiento físico. Asimismo, la rehabilitación biopsicosocial multidisciplinaria también tiene una influencia positiva en el estado laboral en comparación con el tratamiento físico (Kamper et al., 2014).

Investigaciones recientes han evaluado el uso de la educación en neurociencia del dolor para disminuir el dolor y la discapacidad en pacientes sometidos a cirugía lumbar (Louw et al., 2014; Louw et al., 2013; Louw et al., 2015a; Louw et al., 2015b). Varios ensayos controlados aleatorios y una revisión sistemática han demostrado que la neurociencia del dolor tiene un efecto positivo sobre el dolor, la discapacidad, la catastrofización del dolor y el movimiento físico en pacientes con dolor lumbar crónico, extendiéndose dichos resultados hasta 1 año (Louw et al., 2011; Moseley 2004; Moseley 2002; Moseley et al., 2004b; Moseley 2003). En la misma línea, un reciente ensayo controlado aleatorio multicéntrico desarrollado con pacientes sometidos a cirugía por radiculopatía lumbar, demostró que una sesión de neurociencia del dolor preoperatoria complementada con material de lectura obtuvo unos niveles de utilización y costos de atención médica significativamente inferiores, en comparación con los pacientes que recibieron sólo los cuidados habituales, hasta 3 años postcirugía (Louw et al., 2016). Un año después de la operación, el gasto sanitario en los pacientes que recibieron la educación en neurociencia del dolor preoperatoria fue un 45% menor en comparación con los que recibieron la atención habitual. Además, los gastos se mantuvieron un 37% más bajos a los 3 años de la cirugía a pesar de que los niveles de lumbalgia, dolor en las piernas y discapacidad eran similares (Louw et al., 2014; Louw et al., 2016). Estos resultados sugieren un cambio de comportamiento en aquellos pacientes que recibieron la educación en neurociencia del dolor preoperatoria aunque su dolor y discapacidad no mostraron una evolución significativamente diferente a la del grupo control (Louw et al., 2014, Louw et al., 2016).

Las intervenciones en neurociencia del dolor planteadas hasta ahora en los diferentes estudios se basan en la realización de entrevistas personales con el paciente, de unos 30 minutos (Louw et al., 2014, Louw et al., 2016) o 60 minutos de duración (Goudman et al., 2019), complementadas o no con trípticos informativos. En el presente proyecto, nos planteamos la aplicación de sesiones de neurociencia del dolor en un formato audiovisual, a través de vídeos, y complementadas con otras actividades terapéuticas que se describen en los apartados siguientes de esta memoria científico-técnica.

Tratamiento actual de la radiculopatía lumbar

El tratamiento conservador sigue siendo el abordaje inicial de elección para la mayoría de los casos de hernia discal lumbar (França et al. 2018) y de estenosis lumbar (Bagley et al. 2019). La recomendación de las guías clínicas es que las intervenciones no quirúrgicas deben agotarse antes de tomar decisiones sobre la cirugía tanto en los casos de estenosis espinal (Kreiner et al.,2013), como de hernia discal (Kreiner et al., 2014) o espondilolistesis (Watters et al., 009).

Sin embargo, en el caso de pacientes que no mejoran con el tratamiento conservador o que tienen síntomas severos y compresión del saco tecal, generalmente, se recomienda la intervención quirúrgica. El objetivo de la cirugía es descomprimir los elementos neurales comprometidos y aliviar la sintomatología al mismo tiempo que se previene el avance de la degeneración, de una manera que no desestabilice la columna vertebral (Bagley et al. 2019). La cirugía puede aumentar la cantidad de espacio en el canal vertebral a través de la eliminación de porciones de ciertos elementos de la columna vertebral posterior (láminas, facetas, osteofitos, ligamentos, sinovitis o quistes sinoviales); generalmente esto se conoce como 'descompresión' (Zaina et al. 2016).

Las intervenciones clínicas actuales para pacientes con radiculopatía lumbar incluyen ejercicio terapéutico, educación para la salud, terapia de tracción, fisioterapia y tratamiento quirúrgico, (Liu et al., 2019).

El ejercicio terapéutico es uno de los tratamientos conservadores más comunes, que en su mayoría incluyen ejercicios de estabilización de la musculatura del core. Dichos ejercicios permiten entrenar el control neuromuscular, la fuerza y la resistencia de los músculos profundos del tronco, preservando de este modo la estabilidad lumbar. En este sentido, Bayraktar et al. (Bayrakatr et al. 2015) observaron que la realización de un programa de 8 semanas de estabilización de la musculatura del core, realizado tanto dentro como fuera del agua, permitió reducir el dolor y la discapacidad y aumentó la resistencia del tronco y la calidad de vida en pacientes con hernia discal lumbar. En esta misma línea, recientemente, Gaowgzeh et al. (Gaowgzeh et al. 2020) demostraron que la combinación de ejercicios de estabilización del core y terapia descompresiva (tracción) permite mayores beneficios en términos de dolor y discapacidad, en comparación con la realización aislada de dichos ejercicios, en pacientes con hernia discal lumbar.

En el ámbito del ejercicio terapéutico también se han utilizado los ejercicios de control motor como tratamiento conservador de pacientes con hernia discal. Los ejercicios de control motor utilizan principios de aprendizaje motor (fases cognitiva, asociativa y automática) para reentrenar el control de los músculos del tronco, la postura y el patrón de movimiento, lo que conduce a una reducción del dolor y la discapacidad (Macedo et al. 2012). De hecho, en su estudio de 8 semanas de duración, França et al. concluyeron que un programa de entrenamiento de control motor resultó más efectivo que la aplicación de TENS para el alivio del dolor, la reducción de la discapacidad y la mejora de la activación muscular del transverso del abdomen, en pacientes con radiculopatía lumbar (França el al. 2018).

Independientemente de su utilización en el abordaje conservador de la hernia discal, diferentes formas de ejercicio terapéutico han sido utilizadas también en los programas de rehabilitación postoperatoria, con el objetivo de acortar el período de recuperación (Erdogmus et al. 2007) así como mejorar el dolor, la discapacidad y la función física del paciente (Marchand et al., 2016).

En líneas generales, la rehabilitación postquirúrgica se dirige principalmente a ofrecer un soporte y protección lumbar temprano, prevenir la aparición de adherencias de la raíz nerviosa, entrenar la función de los músculos lumbares, así como de los músculos de las extremidades inferiores y prevenir la recurrencia y la reherniación (Chu et al., 2020).

Una revisión de Cochrane de 2009 concluye que el ejercicio intensivo presenta un nivel de evidencia alto en la mejora del estado funcional de los pacientes intervenidos por hernia de disco (Ostelo et al., 2009). De hecho, diferentes estudios observaron mayores beneficios en dolor y discapacidad mediante la aplicación de programas de ejercicios vigorosos o intensos respecto a programas tradicionales o de menor activación (Danielsen et al., 2000, Kjellby-Wendt et al., 2001).

La literatura científica recomienda los programas de ejercicios que comienzan relativamente temprano, de 4 a 6 semanas de la intervención, sin embargo, no existe consenso sobre cuándo comenzar los ejercicios (Ostelo et al., 2009, Erdogmus et al., 2007). Distintos ensayos clínicos aleatorizados han demostrado mayores beneficios de los programas de fisioterapia iniciados tanto 1 semana (Erdogmus et al., 2007) como 6 semanas después de la cirugía (Dolan et al., 2000, Choi et al., 2005), en comparación con un grupo control sin tratamiento (Dolan et al., 2000, Choi et al., 2005) o masaje cervical placebo (Erdogmus et al., 2007). Sin embargo, otros programas de fisioterapia no han podido constatar diferencias en términos de dolor y discapacidad, en comparación con un grupo control con recomendaciones para mantenerse físicamente activo (Mannion et al., 2007). Los programas de fisioterapia implementados en los estudios anteriores se basan, principalmente, en ejercicios dirigidos a mejorar la fuerza, y resistencia de la musculatura abdominal y del tronco, así como la mejora de movilidad de columna y caderas (Dolan et al., 2000), ejercicios de extensión lumbar (Choi et al., 2005) y de estabilización lumbar (Mannion et al., 2007).

Los ejercicios de estabilización dinámica son importantes tanto en el tratamiento conservador de la hernia de disco como en los programas de rehabilitación postoperatoria (Saal et al., 1991). Dichos ejercicios incluyen diferentes técnicas orientadas a la movilidad de la faja abdominal y la adquisición y mantenimiento de una posición lumbar neutral, en la que las fuerzas segmentarias entre el disco y las articulaciones facetarias están mejor equilibradas y la estabilidad lumbar es más eficaz. Los músculos más importantes en este sistema son el multífudus (contribuye significativamente a la estabilidad de la columna protegiendo la región lumbar de movimientos involuntarios y fuerzas de torsión) y el transverso del abdomen (contribuye a la estabilidad lumbar incrementando la presión abdominal) (Demir et al., 2014).

Se ha demostrado que los ejercicios de estabilización lumbar dinámica realizados con supervisión son más efectivos respecto a la realización de ejercicios en el domicilio, en términos de dolor y funcionalidad (Yilmaz et al., 2003). Además, la combinación de ejercicios supervisados de estabilización lumbar con ejercicios en el domicilio (Demir et al., 2014), o con programas de escuela de espalda (Filiz 2005) obtiene mayores beneficios sobre el dolor, discapacidad y movilidad respecto a la realización aislada de ejercicios domiciliarios (Demir 2014) o la combinación de ejercicios domiciliarios y escuela de espalda (Filiz et al. 2005).

La prehabilitación como estrategia terapéutica

El acondicionamiento físico preoperatorio es una estrategia cada vez más común dirigida a mejorar los resultados postoperatorios, incluida la duración del período de hospitalización, la capacidad funcional y las complicaciones perioperatorias (Carli 2005, Lemanu 2013, Valkenet 2011, Ackerman 2004). Además, el período de espera hasta el momento de la cirugía puede representar un momento ideal para preparar al cuerpo de cara a la reducción de movilidad y los cambios físicos que se experimentarán.

Esta estrategia denominada prehabilitación se ha definido como el aumento de la capacidad funcional antes de la cirugía (Carli y Zavorsky 2005), que puede tener un efecto beneficioso sobre el resultado después de la cirugía. Comúnmente, la prehabilitación emplea diferentes modalidades de fisioterapia o ejercicios dirigidos a músculos o articulaciones específicos (Santa Mina et al. 2014). Se ha observado que los programas de entrenamiento preoperatorio pueden mejorar la fuerza de los músculos de la espalda después de la cirugía y regular la función cardiovascular (Chu et al., 2020).

En una revisión sistemática y meta-análisis sobre programas de prehabilitación aplicados en ensayos clínicos aleatorizados de pacientes con diferentes patologías musculoesqueléticas se concluye que, la prehabilitación puede reducir la duración de la hospitalización y, posiblemente, proporcionar beneficios físicos postoperatorios. No obstante, los autores justifican una interpretación cautelosa de estos hallazgos dada la modesta calidad metodológica y el riesgo significativo de sesgo (Santa Mina et al., 2014).

En algunos casos de pacientes con hernia discal se ha observado la aparición de ciertas molestias postoperatorias que podrían ser el resultado de una asociación entre el desacondicionamiento físico del paciente, la presencia de trastornos espinales crónicos y la inactividad impuesta por la propia cirugía (Mannion et al. 2007). Por este motivo, la preparación del paciente antes de la cirugía es un factor fundamental para lograr un óptimo estado de salud que le permita partir de un adecuado nivel funcional.

En la literatura científica actual, la mayoría de referencias sobre el abordaje terapéutico de la hernia de disco se centran en la aplicación de programas de tratamiento conservador, tratamiento quirúrgico o programas de rehabilitación postquirúrgica. Sin embargo, existen pocos estudios que analicen los efectos de programas de prehabilitación en pacientes con cirugías lumbares programadas. Los ensayos controlados aleatorios sugieren que la fisioterapia preoperatoria, así como la educación sobre el dolor, mejoran los resultados en la función y el comportamiento de salud en pacientes con radiculopatía (Louw et al., 2014; Louw et al., 2016; Nielsen et al., 2010).

Los ensayos clínicos aleatorizados realizados hasta el momento, sobre programas de prehabilitación para pacientes que van a ser intervenidos por hernia de disco se basan, principalmente, en la realización de programas de ejercicio supervisado durante un tiempo previo a la intervención, ocho semanas (Nielsen et al. 2010) o nueve semanas (Lindbäck et al. 2018). Sin embargo, también se ha puesto de manifiesto en alguno de estos estudios la importancia de complementar la realización de ejercicio con la educación al paciente sobre neurociencia del dolor. Así pues, Louw et al. concluyen que educar a estos pacientes sobre las respuestas normales a la cirugía lumbar, en el contexto de la neurociencia, puede permitir cambios de comportamiento duraderos después de la cirugía (Louw 2016). Por su parte, Zaina et al. (2016) sugirieron que los tratamientos basados en educación y cognitivo-comportamentales pueden mejorar el dolor y la calidad de vida ofreciendo a los pacientes información sobre su situación y la forma de abordarla, promoviendo de este modo comportamientos saludables (Zaina et al. 2016).

Actualmente no existen pautas para la planificación de la rehabilitación antes de la cirugía de columna lumbar, pero se considera que es importante educar a los pacientes antes de la intervención sobre el ejercicio y las actividades postoperatorias, como el levantamiento de objetos pesados, la acción de agacharse y otras restricciones que debe respetar (Reiter, 2014).

Partiendo de los conocimientos previos y la evidencia científica disponible hasta el momento, el presente Proyecto propone la realización de un programa de prehabilitación que combina la realización de ejercicio terapéutico, la educación sobre los cuidados de la columna vertebral y la educación sobre neurociencia del dolor, en pacientes con dolor lumbar que van a someterse a una intervención quirúrgica, una estrategia terapéutica que hasta el momento no ha sido explorada en esta población.

**METODOLOGÍA**

DISEÑO

Se trata de un ensayo clínico multicéntrico, controlado, aleatorizado, paralelo.

PARTICIPANTES

La muestra del estudio estará compuesta por adultos de entre 18 y 80 años, diagnosticados de radiculopatía lumbar y propuestos para cirugía. Los síntomas serán, predominantemente, dolor en la pierna con o sin déficit neurológico, estando justificada la descompresión quirúrgica. Se excluirá del estudio a los participantes: 1) que se encuentren recibiendo algún otro tratamiento no farmacológico o terapia física para el abordaje de la radiculopatía lumbar, 2) que sean propuestos para cirugía con instrumentación (ej. fusión espinal, artrodesis) 3) que necesiten cirugía inmediata, 4) que padezcan alguna enfermedad con dolor crónico (ej: fibromialgia, síndrome de fatiga crónica) o, 5) síntomas de compresión medular, 6) que hayan sido diagnosticados de tumor maligno o, 7) enfermedad mental, 8) que hayan sido intervenidos previamente de cirugía de columna vertebral y 9) que no tengan posibilidad de acceso a cualquier dispositivo con internet.

El tamaño de la muestra se ha determinado a priori con el programa G-Power 3.1.9.2 (F tests, ANOVA: Repeated measures, within-between interaction). Dicho cálculo se ha obtenido a partir de los resultados de un estudio, de características similares, que evalúa los efectos de un programa de prehabilitación, en pacientes con problemas degenerativos de la columna lumbar (Lindbäck et al., 2018). En este estudio se observó un tamaño del efecto de 0.3 (ɗ Cohen) tras la realización del tratamiento prequirúrgico en la variable principal, Oswestry Disability Index. Por tanto, teniendo en cuenta una probabilidad de alfa= 0.05, una potencia observada= 0.9 y el tamaño del efecto encontrado por Lindbäck (ɗ= 0.3), se ha determinado la necesidad de incluir un total de 82 participantes. Sin embargo, considerando las posibles pérdidas, la muestra se incrementará un 20% requiriéndose finalmente 100 pacientes, 50 por cada grupo.

La muestra se recabará a partir de los Servicios de Cirugía Ortopédica y Traumatología y de Neurocirugía de los Hospitales Arnau de Vilanova y Clínica Universidad de Navarra.

En ningún caso, la inclusión de los pacientes en el estudio supondrá una demora en la programación del quirófano. El periodo estimado de prehabilitación es de 4 semanas. En aquellos casos en los que la indicación de cirugía sea urgente o bien en los casos en los que el paciente desee una cirugía antes del periodo de 4 semanas, se desestimara la inclusión del paciente en el estudio.

PROCEDIMIENTO

El reclutamiento de la muestra se realizará a través de los facultativos especialistas en el tratamiento quirúrgico del paciente con dolor lumbar de los hospitales participantes, quienes valorarán qué pacientes cumplen con los criterios de elegibilidad. Cada centro colaborador informará de la aceptación de participación de un nuevo paciente a la oficina coordinadora, desde donde a través de una lista centralizada se realizará la asignación aleatoria a los grupos de intervención (GI) y grupo control (C). Este dato (grupo) será incluido en un archivo de datos de SPSS, quedando dicha copia en poder exclusivamente de la oficina coordinadora. Para obtener una distribución equilibrada por sexo y edad dentro cada grupo, en la asignación se bloquearán estas variables. Además, se realizará una aleatorización en bloques de 10 participantes para asegurar la igualdad en el número de pacientes en cada grupo, facilitando así la posibilidad de realizar análisis parciales y/o intermedios. Durante el procedimiento de aplicación del programa de intervención y recogida de datos se mantendrá oculta la secuencia de aleatorización, que únicamente conocerá el investigador que realizó la asignación de los pacientes a cada grupo. Así, el resto de investigadores permanecerán ciegos a la asignación de los participantes y se encargarán de la medición de todas las variables pre y post-intervención (inmediatamente tras las 4 semanas de prehabilitación) y seguimiento (1 mes, 6 meses y 1 año después de la cirugía). En todos los casos, y para evitar sesgos por la variabilidad inter-observador, todas las mediciones serán realizadas siempre por los mismos investigadores.

Del mismo modo, los datos sociodemográficos y del resto de variables serán recogidos de forma totalmente anónima gracias a la codificación de cada paciente a partir de la numeración de su historia clínica.

INSTRUMENTOS Y MEDIDAS

A. Datos sociodemográficos y antropométricos básicos: se recabarán datos básicos como sexo, edad, altura, peso, IMC, fumador etc. También se recabará información sobre el nivel educativo, situación laboral, duración de los síntomas, ingesta de medicación y nivel de actividad física en los últimos 12 meses.

B. La percepción de la discapacidad se evaluará mediante el Oswestry Disability Index (ODI) y la versión española validada de Roland– Morris Disability Questionnaire (RMDQ). Varios autores han recomendado el uso de estos dos cuestionarios para evaluar el estado funcional en pacientes con dolor lumbar (Delito et al., 2012; Chapman et al., 2011). El ODI que es una herramienta comúnmente utilizada para medir la discapacidad percibida en pacientes con dolor lumbar (Fairbank y Pinsent, 2000). Este índice contiene 10 elementos. Cada ítem se puntúa de 0 a 5 y la puntuación total se expresa como un porcentaje; las puntuaciones más altas corresponden a una mayor discapacidad. Las puntuaciones porcentuales de ODI de 0 a 20 representan discapacidad mínima, 20 a 40 discapacidad moderada, 40 a 60 discapacidad grave, mientras que las puntuaciones superiores a 60 indican que el paciente está gravemente discapacitado por el dolor (McDowell 2006). El valor de la MCID para ODI, se ha establecido en 10 puntos (de 100) o el 30% de la puntuación inicial (Ostelo et al., 2008). La versión española de RMDQ (Kovacks et al., 2002) consiste en un listado de 24 frases que describen actividades en las que las personas que sufren dolor de espalda suelen estar limitadas. A los sujetos se les pedirá que señalen aquellas frases, únicamente, que describieran su estado en el día de la valoración. A cada frase señalada por el paciente se le otorgará el valor de 1 punto. Por tanto, la puntuación total sobre el grado de discapacidad podrá oscilar entre 0 y 24 puntos. La MCID del cuestionario de discapacidad de Roland Morris ha sido establecida en establecido en 5 puntos (de 24) o el 30% de la puntuación inicial (Ostelo et al., 2008).

C. La percepción del dolor se evaluará mediante la versión española del cuestionario de dolor de McGill (SF-MPQ) (Lázaro et al., 1994) y una escala visual analógica (EVA). El SF-MPQ incluye una serie de adjetivos de entre los cuales, los 11 primeros representan la dimensión sensitiva de la experiencia dolorosa y los adjetivos del 12 al 15, la dimensión afectiva. Cada adjetivo se gradúa en una escala de intensidad de 0= ninguno, 1= leve, 2= moderado, 3= intenso, de manera que el valor total de la experiencia de dolor puede oscilar entre 0 (sin dolor) y 45 puntos (máximo dolor). Se ha demostrado que este cuestionario es una herramienta fiable (Melzack 1987) cuya diferencia mínima clínicamente importante (MCID) se ha establecido en 5 puntos (Strand et al. 2008). Además, se utilizará una EVA consistente en una escala de 0 a 100 mm. con dos extremos etiquetados como "" ningún dolor en absoluto "(puntuación = 0) y "el peor dolor imaginable" (puntuación = 100). La EVA es utilizada ampliamente en la práctica clínica y en estudios de investigación relacionados con el dolor lumbar. La MCID para la EVA es de 15 mm o 30% de la puntuación inicial (utilizando una escala de 100 mm) (Ostelo et al., 2008).

D. La calidad de vida se evaluará mediante la versión validada en español de EuroQol 5D (Badia et al., 1999). Este cuestionario de medición de la calidad de vida relacionada con la salud abarca cinco dimensiones (movilidad, cuidado personal, actividades cotidianas, dolor/malestar y ansiedad/depresión), cada una de las cuales presenta tres niveles de gravedad (sin problemas, algunos problemas o problemas moderados y problemas graves). El cálculo del valor de los diferentes estados de salud se realiza mediante un sistema de codificación establecido, en el que los valores más elevados de puntuación corresponden a niveles más bajos de calidad de vida. El EuroQol 5D ha sido utilizado como indicador de resultados en pacientes con dolor lumbar (Suárez-Almanzor et al., 2000, Lindbäck et al., 2018).

E. Las creencias de temor evitación se analizarán mediante la versión española de Fear Avoidance Behaviour Questionnaire (FABQ) (Kovacs et al., 2006). Este cuestionario consta de dieciséis ítems que el sujeto debe puntuar de 0 a 6 según su grado de conformidad siendo 0= en total desacuerdo, 3=ni de acuerdo ni en desacuerdo y 6=completamente de acuerdo. La puntuación total del cuestionario puede oscilar entre 0 y 96 puntos, reflejando los valores altos un mayor grado de pensamientos de evitación del miedo y los bajos, la ausencia de estas cogniciones. Además, en el cuestionario se identifican dos subescalas. La FABQ-actividad física, está compuesta por cuatro ítems que evalúan cómo podría afectar la realización de actividades físicas cotidianas al dolor experimentado por el paciente. Esta subescala puede tener un rango de 0 a 24 puntos. Por su parte, la FABQ-trabajo está constituida por siete ítems que evalúan cómo podría afectar la realización del trabajo habitual al dolor experimentado por el paciente. Esta subescala puede tener un rango de 0 a 42 puntos.

F. Los niveles de ansiedad y depresión de los participantes se medirán mediante la versión española de Hospital Anxiety and Depression Scale (HADS) (Herrero et al., 2003). La escala HADS revisa los pensamientos y sentimientos que ha experimentado la persona que lo realiza durante los últimos días. Consta de 14 preguntas, donde la ansiedad y la depresión se separan en diferentes categorías, presentando cada trastorno emocional 7 preguntas específicas, con 4 posibles respuestas. Cada una de las respuestas está puntuada de manera diferente de 0 a 3, donde 0 significa que no se experimenta el tipo de problema planteado y 3 que el problema que se plantea se experimenta o siente intensamente. La versión traducida al español ha demostrado buena consistencia interna y validez externa, así como adecuada sensibilidad y especificidad para identificar estos trastornos psiquiátricos.

G. El tiempo de baja laboral: se registrará el tiempo transcurrido desde la baja laboral del paciente hasta su incorporación tras el alta médica, en los casos de pacientes cuyo estatus laboral tenga coherencia con el registro de este dato.

J. El consumo de medicación: se controlará la medicación que consume cada paciente mediante el registro del fármaco utilizado, dosis y tiempo de consumo

H. La utilización de los servicios sanitarios: se registrará si los pacientes han necesitado alguna prueba médica adicional tras la intervención quirúrgica, relacionada específicamente con su cuidado postoperatorio: radiografías, resonancia magnètica, tomografía computarizada, prueba de conducción nerviosa, mielograma y / o otras pruebas médicas. Asimismo, se registrará si los pacientes han recibido algún tratamiento postquirúrgico o si han asistido a consultas con su cirujano de columna; médico de familia; fisioterapeuta; otros médicos especialistas; psicólogo; psiquiatra; y / o otros profesionales sanitarios. En ambos casos (pruebas médicas y proveedores de atención médica), se pedirá a los pacientes que indiquen cuántas veces se realizaron las pruebas o tratamientos. Esta información únicamente será registrada en el seguimiento de 1 año después de la intervención quirúrgica.

I. La satisfacción del paciente con el tratamiento se evaluará mediante la escala Patient Global Impression of Change (PGIC). El paciente indicará mediante esta escala su impresión global de cambio después del programa de prehabilitación. La escala tiene un total de 7 puntos donde 1=completamente recuperado, 2=muy mejorado, 3=ligeramente mejorado, 4 = ningún cambio, 5 = un poco peor, 6 = mucho peor y 7 = enormemente empeorado. Se considerará que ha habido mejoría en las categorías cuya puntuación sea 1,2 ó 3; se considerará que no se han producido cambios con el programa de prehabilitación en la categoría cuya puntuación sea 4; y se considera que ha habido un empeoramiento del paciente en las categorías correspondientes a las puntuaciones 5, 6 y 7. Esta escala ha sido utilizada para la medición de la satisfacción del paciente con dolor crónico lumbar en numerosas ocasiones (Maughan et Lewis 2010, Lindbäck et al., 2018).

Todos los cuestionarios y pruebas se realizarán antes de comenzar el programa de prehabilitación, después del programa de prehabilitación (4 semanas) y en tres momentos del postoperatorio (1 mes, 6 meses y 1 año).

INTERVENCIÓN

Los sujetos incluidos en el grupo de intervención recibirán un programa de prehabilitación que tendrá una duración total de 4 semanas y se desarrollará en base a tres ejes terapéuticos complementarios entre sí: realización de ejercicio terapéutico, educación sobre los cuidados de la columna vertebral y educación en neurociencia del dolor. Todas estas acciones serán desarrolladas por el propio paciente, en el domicilio, a través de la visualización de distintos vídeos.

1.Ejercicio terapéutico

Los participantes realizarán ejercicio terapéutico dirigido al fortalecimiento y control neuromuscular de los músculos abdominales y erectores de columna, principalmente. Los sujetos realizarán diferentes series de ejercicios en distintas posiciones y con dificultad progresivamente creciente y adaptada a su nivel de actividad física. Estos ejercicios se completarán con el fortalecimiento de las extremidades inferiores, estiramientos musculares y la realización de ejercicio aeróbico tal como caminar.

Se realizarán y editarán diferentes vídeos donde se mostrará la realización correcta de los ejercicios. Los vídeos de ejercicio terapéutico contemplarán distintos niveles de dificultad para poderse adaptar a la condición física de todos los participantes. Los sujetos realizarán 5 sesiones semanales de ejercicio terapéutico en el domicilio, guiados por la explicación de los vídeos, durante las 4 semanas previas a la intervención quirúrgica (ver figura 1).

Los pacientes serán controlados semanalmente por un investigador que contactará con ellos para motivarle en la realización de los ejercicios y resolver posibles dudas al respecto.

2.Educación sobre los cuidados de la columna vertebral

El paciente recibirá educación sobre los cuidados de la columna vertebral mediante la visualización de vídeos educativos. Dicho material tendrá como objetivo informar a los pacientes sobre la realización adecuada de movimientos con su columna vertebral y los métodos de protección de la espalda. Incluirán información sobre la estructura y función de la columna vertebral, las principales causas del dolor lumbar y la importancia de los ejercicios. Asimismo, se abordará el autocuidado de la columna mediante ejemplos de posturas adecuadas en la ejecución de tareas habituales (caminar, estar de pie, sentarse, acostarse y levantarse, dormir, levantar objetos pesados, realización de tareas domésticas, posturas laborales, etc.).

Se realizarán y editarán diferentes vídeos, sobre los contenidos monográficos anteriormente mencionados. Los vídeos serán distribuidos a los pacientes de forma progresiva con el fin de pautar su visualización de manera secuencial y lógica. Se instruirá a los pacientes para visualizar estos vídeos a partir de la primera semana y recordar dichos contenidos a lo largo del programa (ver figura 1).

3.Educación sobre neurociencia del dolor

Por último, los sujetos también recibirán educación sobre neurociencia del dolor mediante la visualización de vídeos educativos. Dicho material tendrá como objetivo reconceptualizar el dolor del paciente eliminando la atención sobre la nocicepción de las áreas afectadas y enfocando el dolor como un aumento en la sensibilidad del nervio y la regulación ascendente de los sistemas nerviosos periféricos y centrales. El objetivo será reducir la ansiedad e incertidumbre del paciente y alcanzar expectativas y creencias positivas con respecto a la cirugía programada. Los temas que serán tratados en los diferentes vídeos son la educación sobre fisiología del dolor, la toma de decisión de someterse a la cirugía, los objetivos de la cirugía y la recuperación postoperatoria (Goudman et al., 2019).

Se realizarán y editarán 3 vídeos de unos 15 minutos cada uno, sobre los contenidos monográficos anteriormente mencionados. Los tres vídeos serán distribuidos, de manera secuencial, a lo largo de la última semana del programa de prehabilitación, siguiendo las recomendaciones que se han establecido respecto a la temporalidad idónea de la educación en neurociencia del dolor en el paciente prequirúrgico (Oshodi 2007; Louw et al., 2013) (ver figura 1).


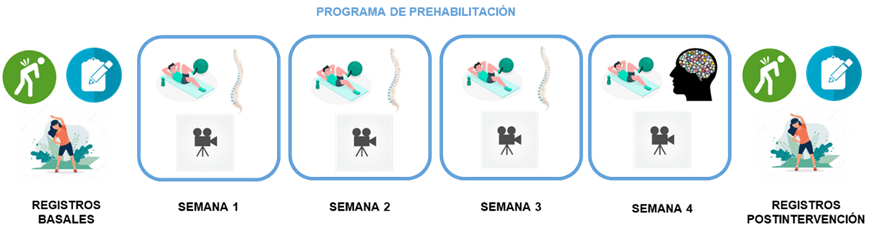


Figura 1: Procedimiento del programa de prehabilitación

Los sujetos incluidos en el grupo control recibirán, por parte del facultativo, información estandarizada sobre el desarrollo de la cirugía y la rehabilitación postquirúrgica, además de la recomendación de mantenerse activo hasta el momento de la intervención quirúrgica.

ANÁLISIS ESTADÍSTICO

En primer lugar, se calcularán los estadísticos descriptivos de los datos sociodemográficos (edad, sexo, altura, peso, IMC, fumador, nivel educativo, situación laboral, duración de los síntomas, nivel de actividad física, etc.) de la muestra y las puntuaciones obtenidas en las diversas medidas.

Antes de realizar los análisis estadísticos se comprobará que en el pretest los dos grupos (control e intervención) están igualados en las variables de estudio y cumplen los supuestos de aplicación de la prueba ANOVA (normalidad, linealidad, homocedasticidad, etc.).

Los efectos de la intervención se compararán respecto al grupo control mediante una prueba de ANOVA mixto considerando como Variables Independientes un Factor entre-sujetos, que sería el Grupo (intervención y control) y un Factor intra-sujetos, el Tiempo, con cuatro niveles: pre-intervención, post-intervención [4 semanas], post-intervención [3 meses] y post-intervención [1 año].

Como medidas de la fuerza del cambio se utilizarán el tamaño de efecto (ηp2) y los intervalos de confianza (IC 95%) para la diferencia de medias.

Asimismo, se llevarán a cabo correlaciones de Pearson para analizar la relación entre las distintas variables: variables de condición de salud (dolor, discapacidad y calidad de vida) y variables comportamentales (creencias de temor evitación, ansiedad y depresión).

En todos los casos el análisis estadístico se realizará por intención de tratar.

Para todo ello, se utilizará el paquete estadístico SPSS 24.0 para Windows (licencia oficial SPSS Inc., Chicago, IL, USA).

**JUSTIFICACIÓN CIENTÍFICA Y ÉTICA**

El presente estudio tiene un diseño de ensayo clínico aleatorizado para evaluar una intervención educativa y rehabilitadora en comparación con el manejo ordinario sobre los resultados de la cirugía lumbar por descompresión nerviosa. La única intervención añadida al manejo habitual de los pacientes en el grupo intervención consiste en recibir un módulo educativo por medio de videos online y la realización de ejercicios autoaplicados de potenciación muscular. Los ejercicios propuestos son de bajo esfuerzo y la literatura previa no ha comunicado efectos secundarios en su realización. Por todo ello, no se esperan efectos secundarios o colaterales derivados de nuestra investigación en la evolución de los pacientes.

Actualmente no existen pautas para la planificación de la rehabilitación antes de la cirugía de columna lumbar, pero se considera que es importante educar a los pacientes antes de la intervención sobre el ejercicio y las actividades postoperatorias. Este estudio propone el desarrollo de un programa de prehabilitación dirigido a pacientes con dolor lumbar, generando un programa coste-efectivo, que pueda ser auto-aplicado en su mayor parte, y que aumente y mantenga la motivación para promover una mejor preparación (tanto física como emocional) de cara a la cirugía, pudiendo desembocar todo ello en una mejor recuperación postquirúrgica y, por tanto, una menor utilización de los servicios sanitarios.

Será requisito imprescindible para la inclusión de los pacientes en el estudio, la lectura comprensiva y posterior firma del consentimiento informado por parte del paciente, acompañada de una adecuada información verbal explicativa del presente estudio de investigación, así como la resolución de las dudas que pudieran surgir. Todos los pacientes serán informados de su derecho y total libertad a poder abandonar el estudio en cualquier momento sin dar explicaciones y sin efectos negativos ni repercusiones sobre su atención médica.

El presente estudio cumple los principios éticos para las investigaciones médicas en seres humanos recogidos en la Declaración de Helsinki de 2013.

**BIBLIOGRAFÍA**

-Ackerman IN, Bennell KL. (2004). Austral J Physiother. 50:25–30.

-Alemano F., Houdayer E., Emedoli, D. (2019). PLoS ONE 14(5): e0216858.

-Al Qaraghli, M. I., & De Jesus, O. (2021). Lumbar Disc Herniation.

-Atlas SJ, Keller RB, Chang Y, Deyo RA, Singer DE. (2001). Spine (Phila Pa 1976); 26:1179–1187.

-Asch HL, Lewis PJ, Moreland DB et al. (2002). J Neurosurg.;96:34–44.

-Bagley C, MacAllister M, Dosselman L et al. Current concepts and recent advances in understanding and managing lumbar spine stenosis. F1000Research 2019, 8(F1000 Faculty Rev):137

-Bayraktar, D., Guclu-Gunduz, A., Lambeck, et al. (2016). Disability and Rehabilitation, 38(12), 1163–1171.

-Brox JI, Storheim K, Grotle M, et al. (2008). Spine J. (6):948-958.

-Carli F, Mayo N. (2001). Br J Anaesth;87:531–3.

- Chad, DA. Lumbar spinalstenosis. Neurologic Clinics. 2007;25(2):407-18.

-Choi, G., Raiturker, P. P., Kim, et al., (2005). Neurosurgery, 57(4), 764–772.

- Danielsen, J. M., Johnsen, R., Kibsgaard, S. K., & Hellevik, E. (2000). Spine, 25(8), 1015–1020.

-Demir, S., Dulgeroglu, D., & Cakci, A. (2014). European Journal of Physical and Rehabilitation Medicine, 50(6), 627–640.

- Deyo RA, Mirza SK, Martin BI, et al.: Trends, major medical complications, and charges associated with surgery for lumbar spinal stenosis in older adults. JAMA. 2010; 303(13): 1259– 65.

- Dolan, P., Greenfield, K., Nelson, R. J., & Nelson, I. W. (2000). Spine, 25(12), 1523–1532

-Erdogmus, C. B., Resch, K.-L., Sabitzer, R., et al. (2007). Spine, 32(19), 2041–2049.

- Filiz, M., Cakmak, A., & Ozcan, E. (2005). Clinical Rehabilitation, 19(1), 4–11.

-Fjeld OR, Grøvle L, Helgeland J, et al. (2019). Bone Joint J;101-B:470-7.

-França, F. J., Nogueira Burke, T., Oliveira Magalhães, et al. (2019). American Journal of Physical Medicine & Rehabilitation, 98(3), 207–214.

-Gadjradj PS, Arts MP, Van Tulder MW, et al. (2017). Spine (Phila Pa 1976).

-Gaowgzeh, R. A. M., Chevidikunnan, M. F., BinMulayh, E. A., & Khan, F. (2020). Journal of Back & Musculoskeletal Rehabilitation, 33(2), 225–231.

-Goudman, L., Huysmans, E., Ickmans, et al. (2019). Physical Therapy, 99(7), 933–945.

-Harvie, D. S. et al. (2015). Psychological Science, 26(4), 385–392.

- Kalichman L, Cole R, Kim DH, et al.: Spinal stenosis prevalence and association with symptoms: the Framingham Study. Spine J. 2009; 9(7): 545–50.

-Kamper SJ, Apeldoorn AT, Chiarotto A, Smeets RJ, et al. (2014). Cochrane Database of Systematic Reviews. 9. Art. No.: CD000963.

- Kenney MP, Milling LS. (2016). Theory, Research, and Practice. 3(3):199-210.

- Kim, S.-S., Min, W.-K., Kim, J.-H., & Lee, B.-H. (2014). Journal of Physical Therapy Science, 26(4), 549–552.

-Klinger R, Geiger F, Schiltenwolf M. (2008). Orthopade.37:1002–1006

- Kreiner DS, ShafferWO, Baisden JL, et al. (2013). Spine J.13:734–43.

-Kreiner DS, Hwang SW, Easa JE et al. (2014). Spine J. 14:180–191.

- Lemanu D, Singh P, MacCormick A, et al. (2013). World J Surg. 37:711–20.

-Liu W, Li Q, Li Z, et al. (2019. Medicine (Baltimore);98: e14682.

-Louw A, Diener I, Butler DS, Puentedura EJ. (2011). Archives of physical medicine and rehabilitation.92(12):2041-2056.

- Louw A, Butler DS, Diener I, et al. (2013). Am J Phys Med Rehabil 92:446-52.

-Louw A, Diener I, Landers MR, Puentedura EJ. (2014). Spine (Phila Pa 1976).39:1449–1457

-Louw A, Diener I, Puentedura EJ. (2015). Int J Spine Surg 9:11.

- Lurie JD, Birkmeyer NJ, Weinstein JN. (2003). Spine. 28(6):616-620.

- Macedo LG, Latimer J, Maher CG, et al. (2012). Phys Ther. 92(3):363-377.

-Maier-Riehle B, Harter M. (2001). Int J Rehabil Res.24:199–206.

- Mannion, A. F., Denzler, R., Dvorak, J., et al. (2007). European Spine Journal: Official Publication of the European Spine Society, the European Spinal Deformity Society, and the European Section of the Cervical Spine Research Society, 16(8), 1101–1117.

-Marchand, A. A., O’Shaughnessy, J., Chatillon, C. E., et al. (2016 Journal of Manipulative and Physiological Therapeutics. 39(9), 668-692.

- Matheve, T., Bogaerts, K., & Timmermans, A. (2020). Journal of Neuroengineering and Rehabilitation, 17(1), 55.

-Mbada Ch., Makinde M., Odole A. Et al. (2019). Human Movement. 20(3): 66– 79

-Melzack R. (2001). J Dent Educ.65:1378-82.

-Moseley L. (2002). Aust J Physiother. 48(4):297-302

-Moseley GL. (2003a). Man Ther.8:130-40.

- Moseley GL. (2003b). Journal of Manual & Manipulative Therapy.11(2):88-94.

-Moseley GL. (2004a). Eur J Pain.8(1):39-45.

-Moseley GL, Nicholas MK, Hodges PW. (2004b). Clin J Pain.20(5):324-330.

-Moseley GL. (2005). Aust J Physiother. 51(1):49-52.

- Nambi, G., Abdelbasset, W. K., Elsayed, et al. (2020). Evidence-Based Complementary and Alternative Medicine: ECAM, 2020, 2981273.

-Nielsen PR, Jorgensen LD, Dahl B, et al. (2010). Clin Rehabil. 24:137–48.

-Nygaard OP, Romner B, Trumpy JH. (1994). Acta Neurochir (Wien). 128:53–56.

-Oosterhuis T, Costa LOP, Maher CG, de Vet HCW et al. (2014). Cochrane database Syst Rev.

- Ostelo RW, Costa LO, Maher CG, de vet HC, van Tulder MW. (2009). Spine. 34:1839-48

- Park, J.-H., Lee, S.-H., & Ko, D.-S. (2013). Journal of Physical Therapy Science, 25(8), 985–988.

-Patil A, Chugh A, Gotecha S, Kotecha M, (2018). J Craniovert Jun Spine; 9:156-62.

-Paulsen, R. T., Rasmussen, J., Carreon, L. Y., & Andersen, M. (2020). The Spine Journal: Official Journal of the North American Spine Society, 20(1), 41–47.

- Rathod TN, Chandanwale A, Ladkat KM, et al. (2014). Indian J Orthop. 48:354–359.

- Reiter, K. (2014). AORN Journal, 99(3), 376-384.

- Saal JA, Saal JS. (1991). Philadelphia: Lea and Febiger; 29: 318–327.

- Santa Mina D, Clarke H, Ritvo P, et al. (2014). Physiotherapy.100:196–207.

-Shen, Y. J., & Huang, S. Z. (2005). Medicare Studies, 1, 85-93.

-Suri P., Pearson A., Scherer E. (2016). PM R. ; 8(5): 405–414.

- Taylor RS, Taylor RJ. (2012). Br J Pain. 6:174–181

- Tomkins-Lane CC, Battié MC, Hu R, et al.: Pathoanatomical characteristics of clinical lumbar pinal stenosis. J Back Musculoskelet Rehabil. 2014; 27(2): 223–9.

- Tomkins-Lane CC, Lafave LM, Parnell JA, Rempel J, Moriartey S, Andreas Y, et al. The spinal stenosis pedometer and nutrition lifestyle intervention (SSPANLI): development and pilot. The

Spine Journal 2015;15(4):577-86.

- Valkenet K, van de Port IG, Dronkers JJ, et al. (2011). Clin Rehabil.25:99–111.

-Voorhies RM, Jiang X, Thomas N. (2007). Spine J. 7:516–524.

-Waddell G.(2004). 2nd ed. (Livingstone C, ed.). Churchill Livingstone

-Wang, R. Q., Lin, Y. H., & Chen, Z. Y. (2016). Physical Therapy, 41(2), 166-167.

-Wang, J., Chen, L., Yu, M., & He, J. (2020). Annals of Palliative Medicine, 9(2), 388–393.

-Watters WC, Bono CM, Gilbert TJ, et al. (2009). Spine J.609–14.

-Watters WC, Baisden J, Gilbert TJ, Kreiner S, Resnick DK,Bono CM, et al. Degenerative lumbar spinal stenosis: an evidence-based clinical guideline for the diagnosis and treatment of degenerative lumbar spinal stenosis. The Spine Journal 2008;8(2):305-10.

-Weinstein JN, Lurie JD, Tosteson TD, et al. (2006). JAMA. 296(20):2451-2459.

- Yílmaz, F., Yílmaz, A., Merdol, F., et al. (2003). Journal of Rehabilitation Medicine, 35(4), 163–167.

- Yilmaz Yelvar, G. D., Çırak, Y., Dalkılınç, M., et al. (2017European Spine Journal : Official Publication of the European Spine Society, the European Spinal Deformity Society, and the European Section of the Cervical Spine Research Society, 26(2), 538–545.

-Yoo, J.-H., Kim, S.-E., Lee, M.-G., et al. (2014). International Journal of Clinical Practice, 68(8), 941–949.

- Zaina F, Tomkins-Lane C, Carragee E, Negrini S. Surgical versus non-surgical treatment for lumbar spinal stenosis. Cochrane Database of Systematic Reviews 2016, Issue 1. Art. No.: CD010264.
