# Supplementary material for: The effects of a prehabilitation programme based on therapeutic exercise, back care education, and pain neuroscience education in patients scheduled for lumbar radiculopathy surgery: A study protocol for a randomised controlled trial
Source: PLoS One. 2024 Jun 6;19(6):e0303979. doi: 10.1371/journal.pone.0303979 (PMC11156268; doi:10.1371/journal.pone.0303979)
Supplement: S2 Data — (DOCX) [file pone.0303979.s002.docx]

Biomedical Research Ethics Committee Application Report

Title: EFFECTIVENESS OF A PREHABILITATION PROGRAM BASED ON THERAPEUTIC EXERCISE, BACK CARE EDUCATION AND PAIN NEUROSCIENCE EDUCATION IN PATIENTS UNDERGOING INTERVENTION FOR LUMBAR RADICULOPATHY

Name of PI research project: María Dolores Arguisuelas Martínez

**TITLE:** EFFICACY OF A PREHABILITATION PROGRAM BASED ON THERAPEUTIC EXERCISE, BACK CARE EDUCATION AND NEUROSCIENCE PAIN EDUCACTION IN PATIENTS SCHEDULED FOR LUMBAR RADICULOPATHY SURGERY**.**

**ABSTRACT:**

Currently, the therapeutic approach of patients who undergo surgery for lumbar radiculopathy is based on post-surgical rehabilitation; however, there are few studies that analyze the effects of prehabilitation programs, that is, programs aimed at improving the patient's preparation and functional capacity before surgery. The present project proposes the evaluation of a prehabilitation program, to be applied during the 4 weeks prior to surgery, based on three complementary therapeutic axes: therapeutic exercise, education on spinal care and education on the neuroscience of pain. The effects on the patient's health condition variables (pain, disability and quality of life) and behavioral variables (fear-avoidance beliefs, catastrophism, anxiety and depression) will be evaluated. In conclusion, we propose the development of a prehabilitation program aimed at patients with low back pain, generating a cost-effective program that can be self-applied for the most part, and that increases and maintains motivation to promote better preparation (both physical and emotional) for surgery, which could lead to a better post-surgical recovery and, therefore, a lower use of health services.

**INTRODUCTION AND BACKGROUND:**

Relevance of low back pain as a public health problem: impact on disability and quality of life.

Low back pain is one of the most prevalent chronic pain conditions worldwide, affecting more than 70% of the general population throughout life, with an annual incidence of 40%. Moreover, it shows a high tendency to become chronic or produce recurrences that can severely limit patients' daily lives (Balague et al., 2012). In a survey carried out in the Spanish population, the one-year prevalence was 20% for low back pain (Fernández de las Peñas, 2011). It is currently the most frequent reason for sick leave and the main cause of disability in people under 45 years of age. The economic cost attributable to LBP is 8945.6 million euros per year, representing 0.68% of the Spanish Gross Domestic Product (Alonso-García 2020). In our country, low back pain is the first cause of disability adjusted for life years, surpassing heart disease (Soriano, 2018).

Spinal symptomatology and function can be improved by rehabilitation in most patients, however, 10% to 20% of patients require surgery (Liu et al., 2019). The acute postoperative period is associated with a marked reduction in physical function and health-related quality of life (Carli and Mayo 2001). Postoperative hospitalization usually lasts 1-3 days and it is common to begin physical rehabilitation 4-6 weeks after surgery (Oosterhuis et al., 2017). Approximately 80% of patients return to work 12 months after surgery (Nygaard et al., 1994). The reasons are varied and include patient selection, psychosocial aspects, and variability in the spectrum of degenerative disc disease, although postoperative rehabilitation also has an important influence on surgical outcomes (Choi et al. 2005). Recently, however, Paulsen et al. observed that referral to a supervised individualized rehabilitation group or a home-based group without exercise planning does not affect the duration of postoperative sick leave, return to work or work capacity in patients recovering after lumbar disc herniation surgery. In contrast, they did find a significant association between the duration of radiating leg pain and work capacity during the preoperative phase with the duration of sick leave (Paulsen et al., 2020).

Lumbar disc herniation is a common cause of pain and disability in the general population (Gadjradj et al., 2017). In fact, in patients with lumbar radiculopathy, it has been estimated that a percentage between 10% and 40% may experience persistent pain and disability even after discectomy (Lurie et al., 2003; Weinstein et al., 2006). Disability and quality of life are interrelated and affect each other. Therefore, managing disability can promote health, thereby improving quality of life (Chu et al., 2020). Similarly, regardless of etiology, lumbar stenosis can cause chronic pain and disability, dramatically reducing quality of life, mobility and function (Chad 2007).

The success rate of surgery for lumbar radiculopathy interventions is estimated to be between 75% and 80% (Atlas et al., 2001, Asch et al., 2002). Despite the high surgical success rate, 23% to 28% of patients undergoing decompression surgery end up with chronic back or leg pain (Atlas et al., 2001, Voorhies et al., 2007, Kreiner et al., 2014). These unfavorable outcomes are associated with higher levels of postoperative health care utilization, incurring higher health care costs (Taylor and Taylor 2012) and resulting in a high socioeconomic burden.

The development of chronic post-surgical pain may be due to a multitude of factors, including but not limited to negative psychological factors, pre-existing chronic pain, psychological disorders, high C-reactive protein sensitivity or preoperative joint pain (Voorhies et al., 2007, Klinger et al., 2008, Rathod et al., 2014). Thus, it appears that the patient's experience of pain in addition to the individual's attributes towards pain play a very important role in the recovery from this condition. For this reason, one of the main axes of the intervention proposed in the present Project is based on educating the patient about pain neuroscience, with the aim of reducing the fear associated with low back pain, by providing information to the patient about the neurophysiology, processing and representation of the pain experience.

On the other hand, taking into account that postoperative rehabilitation has shown a limited effect in the reduction of pain and disability, it is necessary to consider other strategies different from those that have been developed so far, which may have an impact on these health conditions. This is why the present project proposes a preoperative intervention aimed at improving the patient's functional capacity before surgery and his or her coping strategies before surgery.

Pain Neuroscience

In recent decades, the biopsychosocial model has been applied as a framework for understanding the complexity of chronic pain in general, and low back pain in particular, in preference to a purely biomedical approach. From this perspective, many factors, including biophysical, psychological, social and genetic factors, and comorbidities can contribute to disability in low back pain.

However, currently, in the context of the low back pain patient facing surgery, the preoperative advice the patient receives is mainly focused on biomechanical and anatomical models for the approach to pain and disability (Brox et al. 2008, Moseley 2004). This type of information has not only shown limited efficacy, but may even increase fears, anxiety and stress in patients with lumbar radiculopathy who are going to receive a surgical intervention (Maier-Riehle et Harter 2001, Louw et al., 2014).

Recent research on educational strategies for patients with low back pain shows an increase in the use of therapeutic neuroscience education. This aims to reduce pain and disability by helping patients gain a greater understanding of the biological aspects and physiological processes involved in their pain experience. Pain neuroscience is a cognitive-based intervention that differs from traditional education strategies in that it does not focus on anatomical or biomechanical aspects, but rather on neurophysiology, neurobiology, processing and representation, and meaning of pain. In short, pain neuroscience de-emphasizes traditional anatomical tissue-based models of pain (Moseley 2003a, Melzack 2001), and aims to reduce the fear associated with low back pain by providing more information about the pain and neurophysiology of a pain experience.

A systematic review, conducted by Louw et al. (2011), suggests strong evidence for pain neuroscience education on pain, disability, and physical performance in musculoskeletal pain, particularly in spinal disorders. Specifically, the effects observed were a decrease in fear and changes in pain perception (Moseley 2003b); an immediate effect on pain attitudes (Moseley 2003a); improvements in pain, cognition, and physical performance (Moseley 2004a); increased pain thresholds during physical tasks (Moseley et al., 2004b); improved therapeutic exercise performance (Moseley 2002); and significantly reduced brain activity characteristic of pain experience (Moseley 2005).

On the other hand, a Cochrane review conducted in patients with chronic low back pain concluded that patients who receive multidisciplinary biopsychosocial rehabilitation probably experience less pain and disability than those who receive usual care or physical treatment. Likewise, multidisciplinary biopsychosocial rehabilitation also has a positive influence on work status compared to physical treatment (Kamper et al., 2014).

Recent research has evaluated the use of pain neuroscience education to decrease pain and disability in patients undergoing low back surgery (Louw et al., 2014; Louw et al., 2013; Louw et al., 2015a; Louw et al., 2015b). Several randomized controlled trials and a systematic review have demonstrated that pain neuroscience has a positive effect on pain, disability, pain catastrophizing, and physical movement in patients with chronic low back pain, with such results extending up to 1 year (Louw et al., 2011; Moseley 2004; Moseley 2002; Moseley 2002; Moseley et al., 2004b; Moseley 2003). Along the same lines, a recent multicenter randomized controlled trial developed with patients undergoing surgery for lumbar radiculopathy demonstrated that a preoperative pain neuroscience session supplemented with reading material obtained significantly lower utilization levels and health care costs, compared to patients receiving usual care alone, up to 3 years post-surgery (Louw et al., 2016). One year postoperatively, healthcare spending in patients who received preoperative pain neuroscience education was 45% lower compared to those who received usual care. Furthermore, expenditures remained 37% lower 3 years after surgery despite similar levels of low back pain, leg pain, and disability (Louw et al., 2014; Louw et al., 2016). These results suggest a behavioral change in those patients who received preoperative pain neuroscience education although their pain and disability did not show a significantly different evolution than the control group (Louw et al., 2014, Louw et al., 2016).

The pain neuroscience interventions proposed so far in the different studies are based on personal interviews with the patient, lasting about 30 minutes (Louw et al., 2014, Louw et al., 2016) or 60 minutes (Goudman et al., 2019), complemented or not with informative triptychs. In the present project, we consider the application of pain neuroscience sessions in an audiovisual format, through videos, and complemented with other therapeutic activities described in the following sections of this scientific-technical report.

Current treatment of lumbar radiculopathy.

Conservative treatment remains the initial approach of choice for most cases of lumbar disc herniation (França et al. 2018) and lumbar stenosis (Bagley et al. 2019). The recommendation from clinical guidelines is that non-surgical interventions should be exhausted before decisions are made about surgery in both cases of spinal stenosis (Kreiner et al.,2013), disc herniation (Kreiner et al., 2014) or spondylolisthesis (Watters et al., 009).

However, in the case of patients who do not improve with conservative treatment or who have severe symptoms and compression of the thecal sac, surgical intervention is generally recommended. The goal of surgery is to decompress the involved neural elements and relieve symptomatology while preventing progression of degeneration in a manner that does not destabilize the spine (Bagley et al. 2019). Surgery can increase the amount of space in the spinal canal through the removal of portions of certain posterior spinal elements (laminae, facets, osteophytes, ligaments, synovitis, or synovial cysts); this is generally referred to as 'decompression' (Zaina et al. 2016).

Current clinical interventions for patients with lumbar radiculopathy include therapeutic exercise, health education, traction therapy, physical therapy, and surgical treatment, (Liu et al., 2019).

Therapeutic exercise is one of the most common conservative treatments, most of which include core muscle stabilization exercises. Such exercises allow training neuromuscular control, strength and endurance of the deep trunk muscles, thus preserving lumbar stability. In this regard, Bayraktar et al. (Bayrakatr et al. 2015) observed that the performance of an 8-week program of core muscle stabilization, performed both in and out of the water, led to reduced pain and disability and increased trunk endurance and quality of life in patients with lumbar disc herniation. In the same line, recently, Gaowgzeh et al. (Gaowgzeh et al. 2020) demonstrated that the combination of core stabilization exercises and decompressive therapy (traction) allows greater benefits in terms of pain and disability, compared to the isolated performance of such exercises, in patients with lumbar disc herniation.

In the field of therapeutic exercise, motor control exercises have also been used as a conservative treatment for patients with herniated discs. Motor control exercises use motor learning principles (cognitive, associative and automatic phases) to retrain trunk muscle control, posture and movement pattern, leading to a reduction in pain and disability (Macedo et al. 2012). In fact, in their 8-week study, França et al. concluded that a motor control training program was more effective than TENS application for pain relief, disability reduction and improvement of transverse abdominis muscle activation, in patients with lumbar radiculopathy (França el al. 2018).

Regardless of their use in the conservative approach to disc herniation, different forms of therapeutic exercise have also been used in postoperative rehabilitation programs, with the aim of shortening the recovery period (Erdogmus et al. 2007) as well as improving the patient's pain, disability and physical function (Marchand et al., 2016).

Generally speaking, post-surgical rehabilitation is mainly aimed at providing early lumbar support and protection, preventing the occurrence of nerve root adhesions, training the function of lumbar muscles as well as lower extremity muscles, and preventing recurrence and reherniation (Chu et al., 2020).

A Cochrane review of 2009 concludes that intensive exercise presents a high level of evidence in the improvement of the functional status of patients operated for disc herniation (Ostelo et al., 2009). In fact, different studies observed greater benefits in pain and disability through the application of vigorous or intense exercise programs compared to traditional or lower activation programs (Danielsen et al., 2000, Kjellby-Wendt et al., 2001).

The scientific literature recommends exercise programs starting relatively early, 4 to 6 weeks after surgery, however, there is no consensus on when to start exercises (Ostelo et al., 2009, Erdogmus et al., 2007). Different randomized clinical trials have demonstrated greater benefits of physical therapy programs initiated both 1 week (Erdogmus et al., 2007) and 6 weeks after surgery (Dolan et al., 2000, Choi et al., 2005), compared to a no-treatment control group (Dolan et al., 2000, Choi et al., 2005) or placebo cervical massage (Erdogmus et al., 2007). However, other physical therapy programs have failed to find differences in terms of pain and disability compared to a control group with recommendations to be physically active (Mannion et al., 2007). The physiotherapy programs implemented in previous studies are mainly based on exercises aimed at improving the strength and endurance of the abdominal and trunk musculature, as well as improving the mobility of the spine and hips (Dolan et al., 2000), lumbar extension exercises (Choi et al., 2005) and lumbar stabilization (Mannion et al., 2007).

Dynamic stabilization exercises are important both in the conservative treatment of disc herniation and in postoperative rehabilitation programs (Saal et al., 1991). These exercises include different techniques aimed at the mobility of the abdominal girdle and the acquisition and maintenance of a neutral lumbar position, in which the segmental forces between the disc and the facet joints are better balanced and lumbar stability is more effective. The most important muscles in this system are the multifudus (contributes significantly to spinal stability by protecting the lumbar region from involuntary movements and twisting forces) and the transverse abdominis (contributes to lumbar stability by increasing abdominal pressure) (Demir et al., 2014).

It has been shown that dynamic lumbar stabilization exercises performed with supervision are more effective than home exercises in terms of pain and functionality (Yilmaz et al., 2003). Furthermore, the combination of supervised lumbar stabilization exercises with home exercises (Demir et al., 2014), or with back school programs (Filiz 2005) obtains greater benefits on pain, disability and mobility with respect to the isolated performance of home exercises (Demir 2014) or the combination of home exercises and back school (Filiz et al. 2005).

Prehabilitation as a therapeutic strategy

Preoperative physical conditioning is an increasingly common strategy aimed at improving postoperative outcomes, including length of hospitalization period, functional capacity and perioperative complications (Carli 2005, Lemanu 2013, Valkenet 2011, Ackerman 2004). In addition, the waiting period until the time of surgery may represent an ideal time to prepare the body for the reduced mobility and physical changes that will be experienced.

This strategy called prehabilitation has been defined as increasing functional capacity prior to surgery (Carli and Zavorsky 2005), which can have a beneficial effect on outcome after surgery. Commonly, prehabilitation employs different physical therapy modalities or exercises targeting specific muscles or joints (Santa Mina et al. 2014). It has been observed that preoperative training programs can improve back muscle strength after surgery and regulate cardiovascular function (Chu et al., 2020).

A systematic review and meta-analysis of prehabilitation programs applied in randomized clinical trials of patients with different musculoskeletal pathologies concludes that prehabilitation can reduce the duration of hospitalization and possibly provide postoperative physical benefits. However, the authors justify a cautious interpretation of these findings given the modest methodological quality and significant risk of bias (Santa Mina et al., 2014).

In some cases of patients with disc herniation, the appearance of certain postoperative discomfort has been observed, which could be the result of an association between the patient's physical deconditioning, the presence of chronic spinal disorders and the inactivity imposed by the surgery itself (Mannion et al. 2007). For this reason, patient preparation prior to surgery is a fundamental factor in achieving an optimal state of health that allows the patient to start from an adequate functional level.

In the current scientific literature, most references on the therapeutic approach to disc herniation focus on the application of conservative treatment programs, surgical treatment or post-surgical rehabilitation programs. However, there are few studies that analyze the effects of prehabilitation programs in patients with scheduled lumbar surgery. Randomized controlled trials suggest that preoperative physical therapy, as well as pain education, improve outcomes in function and health behavior in patients with radiculopathy (Louw et al., 2014; Louw et al., 2016; Nielsen et al., 2010).

The randomized clinical trials conducted so far on prehabilitation programs for patients undergoing surgery for herniated discs are mainly based on supervised exercise programs for eight weeks (Nielsen et al. 2010) or nine weeks (Lindbäck et al. 2018) prior to surgery. However, the importance of complementing exercise performance with patient education on pain neuroscience has also been highlighted in some of these studies. Thus, Louw et al. conclude that educating these patients about normal responses to low back surgery, in the context of neuroscience, may allow for lasting behavioral changes after surgery (Louw 2016). For their part, Zaina et al. (2016) suggested that education-based and cognitive-behavioral treatments can improve pain and quality of life by providing patients with information about their situation and how to address it, thereby promoting healthy behaviors (Zaina et al. 2016).

Currently there are no guidelines for rehabilitation planning before lumbar spine surgery, but it is considered important to educate patients before the intervention about exercise and postoperative activities, such as heavy lifting, bending action and other restrictions to be respected (Reiter, 2014).

Based on previous knowledge and the scientific evidence available so far, the present Project proposes the implementation of a prehabilitation program that combines therapeutic exercise, back care education and pain neuroscience education, in patients scheduled for lumbar radiculopthy surgery, a therapeutic strategy that has not been explored in this population so far.

**METHODOLOGY**

DESIGN

This is a multicenter, controlled, randomized, parallel, controlled clinical trial.

PARTICIPANTS

The study sample will be composed of adults between 18 and 80 years of age, diagnosed with lumbar radiculopathy and proposed for surgery. The symptoms will be predominantly leg pain with or without neurological deficit, and surgical decompression will be justified. Participants will be excluded from the study: 1) who are receiving any other non-pharmacological treatment or physical therapy for the treatment of lumbar radiculopathy, 2) who are proposed for surgery with instrumentation (e.g., spinal fusion, arthrodesis, arthrodesis), 3) who are not receiving any other treatment for lumbar radiculopathy and who are not receiving any other treatment. 3) they are in need of acute surgery 4) they suffer from a chronic pain condition (e.g., fibromyalgia, chronic fatigue syndrome), or 5) exhibit symptoms of spinal cord compression, 6) have been diagnosed with a malignant tumor, or 7) have a mental illness, 8) have undergone previous spinal surgery, and 9] do not have access to any internet-enabled device.

The sample size was determined a priori with the G-Power 3.1.9.2 program (F tests, ANOVA: Repeated measures, within-between interaction). Such calculation was obtained from the results of a study, with similar characteristics, evaluating the effects of a prehabilitation program, in patients with degenerative lumbar spine problems (Lindbäck et al., 2018). In this study, an effect size of 0.3 (ɗ Cohen) was observed after completion of the presurgical treatment on the primary variable, Oswestry Disability Index. Therefore, taking into account a probability of alpha= 0.05, an observed power= 0.9 and the effect size found by Lindbäck (ɗ= 0.3), the need to include a total of 82 participants has been determined. However, considering the possible losses, the sample will be increased by 20%, finally requiring 100 patients, 50 for each group.

The sample will be collected from the Orthopedic Surgery and Traumatology and Neurosurgery Departments of the Arnau de Vilanova and Clínica Universidad de Navarra Hospitals.

In no case will the inclusion of patients in the study entail a delay in the scheduling of the operating room. The estimated prehabilitation period is 4 weeks. In those cases in which the indication for surgery is urgent or in those cases in which the patient desires surgery before the 4-week period, the patient's inclusion in the study will be rejected.

PROCEDURE

The sample recruitment will be carried out through specialist physicians in the surgical treatment of patients with lumbar pain from participating hospitals. These specialists will assess which patients meet the eligibility criteria. Each collaborating center will report the acceptance of a new patient's participation to the coordinating office. Through a centralized list, random assignment to the intervention group (IG) and the control group (CG) will be conducted. This information (group) will be included in an SPSS data file, with the exclusive possession of the coordinating office. To achieve a balanced distribution by gender and age within each group, these variables will be blocked during assignment. Additionally, randomization in blocks of 10 participants will be performed to ensure equality in the number of patients in each group, facilitating the possibility of partial and/or interim analyses.

During the implementation of the intervention program and data collection procedure, the randomization sequence will be kept hidden, known only to the researcher who assigned patients to each group. Thus, the remaining researchers will remain blind to the participants' assignment and will be responsible for measuring all pre- and post-intervention variables (immediately after the 4 weeks of prehabilitation) and follow-up (1 month, 6 months, and 1 year after surgery). In all cases, and to avoid biases due to inter-observer variability, all measurements will be consistently performed by the same researchers.

Similarly, sociodemographic and other variable data will be collected anonymously through the encoding of each patient based on their medical record numbering.

INSTRUMENTS AND MEASURES

A. Basic sociodemographic and anthropometric data: basic data such as sex, age, height, weight, BMI, smoker, etc. will be collected. Information will also be collected on educational level, employment status, duration of symptoms, medication intake, and level of physical activity in the last 12 months.

B. The perception of disability will be evaluated using the Oswestry Disability Index (ODI) and the validated Spanish version of the Roland-Morris Disability Questionnaire (RMDQ). Several authors have recommended the use of these two questionnaires to evaluate functional status in patients with low back pain (Delito et al., 2012; Chapman et al., 2011). The ODI is a commonly used tool to measure perceived disability in patients with low back pain (Fairbank and Pinsent, 2000). This index contains 10 elements. Each item is scored from 0 to 5 and the total score is expressed as a percentage; Higher scores correspond to greater disability. ODI percentage scores of 0 to 20 represent minimal disability, 20 to 40 moderate disability, 40 to 60 severe disability, while scores above 60 indicate that the patient is severely disabled by pain (McDowell 2006). The MCID value for ODI has been established at 10 points (out of 100) or 30% of the initial score (Ostelo et al., 2008). The Spanish version of RMDQ (Kovacks et al., 2002) consists of a list of 24 sentences that describe activities in which people who suffer from back pain are usually limited. Subjects will be asked to indicate only those phrases that describe their state on the day of the assessment. Each phrase indicated by the patient will be given the value of 1 point. Therefore, the total score on the degree of disability may range between 0 and 24 points. The MCID of the Roland Morris Disability Questionnaire has been established at 5 points (out of 24) or 30% of the initial score (Ostelo et al., 2008).

C. Pain perception will be evaluated using the Spanish version of the McGill Pain Questionnaire (SF-MPQ) (Lázaro et al., 1994) and a visual analogue scale (VAS). The SF-MPQ includes a series of adjectives, among which the first 11 represent the sensitive dimension of the painful experience and adjectives 12 to 15, the affective dimension. Each adjective is graded on an intensity scale of 0 = none, 1 = mild, 2 = moderate, 3 = intense, so that the total value of the pain experience can range between 0 (no pain) and 45 points (maximum). pain). This questionnaire has been shown to be a reliable tool (Melzack 1987) whose minimum clinically important difference (MCID) has been established at 5 points (Strand et al. 2008). Additionally, an EVA consisting of a scale of 0 to 100 mm will be used. with two extremes labeled ""no pain at all" (score = 0) and "worst pain imaginable" (score = 100). The VAS is widely used in clinical practice and in research studies related to low back pain. The MCID for the VAS is 15 mm or 30% of the baseline score (using a 100 mm scale) (Ostelo et al., 2008).

D. Quality of life will be assessed using the validated Spanish version of EuroQol 5D (Badia et al., 1999). This questionnaire measuring health-related quality of life covers five dimensions (mobility, personal care, activities of daily living, pain/discomfort, and anxiety/depression), each of which presents three levels of severity (no problems, some problems or moderate problems and serious problems). The calculation of the value of different health states is carried out using an established coding system, in which higher score values correspond to lower levels of quality of life. The EuroQol 5D has been used as an outcome indicator in patients with low back pain (Suárez-Almanzor et al., 2000, Lindbäck et al., 2018).

E. Fear avoidance beliefs will be analyzed using the Spanish version of the Fear Avoidance Behavior Questionnaire (FABQ) (Kovacs et al., 2006). This questionnaire consists of sixteen items that the subject must score from 0 to 6 according to their degree of agreement, with 0 = completely disagree, 3 = neither agree nor disagree and 6 = completely agree. The total score of the questionnaire can range between 0 and 96 points, with high values reflecting a greater degree of fear-avoidance thoughts and low values reflecting the absence of these cognitions. Additionally, two subscales are identified in the questionnaire. The FABQ-physical activity is composed of four items that evaluate how performing daily physical activities could affect the pain experienced by the patient. This subscale can have a range from 0 to 24 points. For its part, the FABQ-work is made up of seven items that evaluate how performing regular work could affect the pain experienced by the patient. This subscale can have a range from 0 to 42 points.

F. The anxiety and depression levels of the participants will be measured using the Spanish version of the Hospital Anxiety and Depression Scale (HADS) (Herrero et al., 2003). The HADS scale reviews the thoughts and feelings that the person taking it has experienced during the last few days. It consists of 14 questions, where anxiety and depression are separated into different categories, each emotional disorder presenting 7 specific questions, with 4 possible answers. Each of the responses is scored differently from 0 to 3, where 0 means that the type of problem posed is not experienced and 3 means that the problem posed is experienced or felt intensely. The version translated into Spanish has demonstrated good internal consistency and external validity, as well as adequate sensitivity and specificity to identify these psychiatric disorders.

G. The time of sick leave: the time elapsed from the patient's sick leave to their incorporation after medical discharge will be recorded, in the cases of patients whose work status is consistent with the recording of this data.

J. Medication intake: the medication consumed by each patient will be controlled by recording the drug used, dose and time of consumption H. Use of health services: whether patients have needed any additional medical tests after the intervention will be recorded surgery, specifically related to your postoperative care: x-rays, MRI, CT scan, nerve conduction test, myelogram and/or other medical tests. Likewise, it will be recorded if the patients have received any post-surgical treatment or if they have attended consultations with their spine surgeon; family doctor; physiotherapist; other medical specialists; psychologist; psychiatrist; and/or other healthcare professionals. In both cases (medical tests and healthcare providers), patients will be asked to indicate how many times the tests or treatments were performed. This information will only be recorded in the 1-year follow-up after the surgical intervention.

I. Patient satisfaction with treatment will be evaluated using the Patient Global Impression of Change (PGIC) scale. Using this scale, the patient will indicate his or her global impression of change after the prehabilitation program. The scale has a total of 7 points where 1 = completely recovered, 2 = much improved, 3 = slightly improved, 4 = no change, 5 = a little worse, 6 = much worse and 7 = greatly worsened. It will be considered that there has been improvement in the categories whose score is 1,2 or 3; It will be considered that no changes have occurred with the prehabilitation program in the category whose score is 4; and it is considered that there has been a worsening of the patient in the categories corresponding to scores 5, 6 and 7. This scale has been used to measure the satisfaction of patients with chronic low back pain on numerous occasions (Maughan et Lewis 2010, Lindbäck et al., 2018).

All questionnaires and tests will be carried out before starting the prehabilitation program, after the prehabilitation program (4 weeks) and at three postoperative moments (1 month, 6 months and 1 year).

INTERVENTION

The subjects included in the intervention group will receive a prehabilitation program that will last a total of 4 weeks and will be developed based on three therapeutic axes that complement each other: therapeutic exercise, education on spinal care and education in neuroscience of pain. All these actions will be carried out by the patient himself, at home, through the viewing of different videos.

1.Therapeutic exercise

Participants will perform therapeutic exercise aimed at strengthening and neuromuscular control of the abdominal muscles and erector spinae, mainly. Subjects will perform different series of exercises in different positions and with progressively increasing difficulty adapted to their level of physical activity. These exercises will be completed with strengthening of the lower extremities, muscle stretching and performing aerobic exercise such as walking.

Different videos will be made and edited showing the correct performance of the exercises. The therapeutic exercise videos will include different levels of difficulty in order to adapt to the physical condition of all participants. Subjects will perform 5 weekly sessions of therapeutic exercise at home, guided by the explanation of the videos, during the 4 weeks prior to the surgical intervention (see figure 1). Patients will be monitored weekly by a researcher who will contact them to motivate them to perform the exercises and resolve any doubts regarding them.

2. Back care education

The patient will receive education on spinal care by watching educational videos. This material will aim to inform patients about the proper execution of movements with their spine and back protection methods. They will include information about the structure and function of the spine, the main causes of low back pain, and the importance of exercises. Likewise, self-care of the spine will be addressed through examples of appropriate postures in the execution of common tasks (walking, standing, sitting, lying down and getting up, sleeping, lifting heavy objects, performing household tasks, work postures, etc.). Different videos will be made and edited on the monographic content mentioned above. The videos will be distributed to patients progressively in order to guide their viewing in a sequential and logical manner. Patients will be instructed to watch these videos from the first week and remember these contents throughout the program (see figure 1).

3. Pain neuroscience education

Lastly, subjects will also receive education on the neuroscience of pain through viewing educational videos. Such material will aim to reconceptualize the patient's pain by removing attention on nociception of the affected areas and focusing on pain as an increase in nerve sensitivity and upregulation of the peripheral and central nervous systems. The objective will be to reduce the patient's anxiety and uncertainty and achieve positive expectations and beliefs regarding the scheduled surgery. The topics that will be covered in the different videos are education on the physiology of pain, making the decision to undergo surgery, the objectives of surgery and postoperative recovery (Goudman et al., 2019).

Tree videos of about 15 minutes each will be made and edited on the monographic content mentioned above. The three videos will be distributed, sequentially, throughout the last week of the prehabilitation program, following the recommendations that have been established regarding the ideal timing of education in pain neuroscience in the presurgical patient (Oshodi 2007; Louw et al., 2013) (see figure 1).


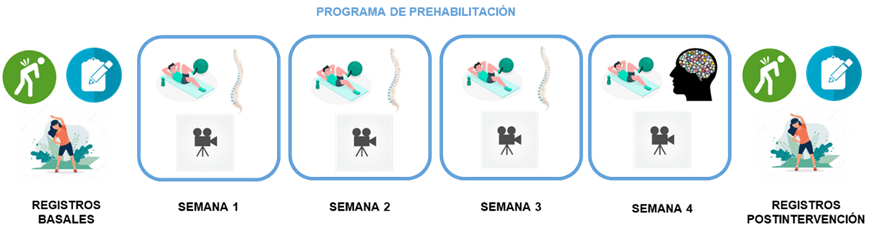


Figure 1: Prehabilitation program procedure

Subjects included in the control group will receive, from the physician, standardized information about the course of surgery and post-surgical rehabilitation, in addition to the recommendation to remain active until the moment of surgery.

STATISTICAL ANALYSIS

Firstly, the descriptive statistics of the sociodemographic data (age, sex, height, weight, BMI, smoker, educational level, employment status, duration of symptoms, level of physical activity, etc.) of the sample and the scores obtained in the various measures.

Before carrying out the statistical analyzes it will be verified that in the pretest the two groups (control and intervention) are equal in the study variables and meet the assumptions of application of the ANOVA test (normality, linearity, homoscedasticity, etc.).

The effects of the intervention will be compared with the control group using a mixed ANOVA test considering as Independent Variables a between-subjects Factor, which would be Group (intervention and control) and an intra-subjects Factor, Time, with four levels: pre-intervention, post-intervention [4 weeks], post-intervention [3 months], and post-intervention [1 year]. As measures of the strength of the change, the effect size (ηp2) and the confidence intervals (95% CI) for the difference in means will be used. Likewise, Pearson correlations will be carried out to analyze the relationship between the different variables: health condition variables (pain, disability and quality of life) and behavioral variables (beliefs of fear, avoidance, anxiety and depression). In all cases the statistical analysis will be performed by intention to treat.

For all this, the SPSS 24.0 statistical package for Windows will be used (official license SPSS Inc., Chicago, IL, USA).

**SCIENTIFIC AND ETHICAL JUSTIFICATION**

The present study has a randomized clinical trial design to evaluate an educational and rehabilitative intervention in comparison with ordinary management on the results of lumbar surgery for nerve decompression. The only intervention added to the usual management of patients in the intervention group consists of receiving an educational module through online videos and performing self-applied muscle strengthening exercises. The proposed exercises are low effort and previous literature has not reported side effects when performing them. For all these reasons, no side or collateral effects derived from our research on the evolution of patients are expected.

There are currently no guidelines for rehabilitation planning before lumbar spine surgery, but it is considered important to educate patients preoperatively about exercise and postoperative activities. This study proposes the development of a prehabilitation program aimed at patients with low back pain, generating a cost-effective program that can be self-applied for the most part, and that increases and maintains motivation to promote better preparation (both physical as emotional) in the face of surgery, all of which can lead to better post-surgical recovery and, therefore, less use of health services.

It will be an essential requirement for the inclusion of patients in the study, the comprehensive reading and subsequent signing of the informed consent by the patient, accompanied by adequate verbal information explaining the present research study, as well as the resolution of any doubts that may arise. All patients will be informed of their right and complete freedom to withdraw from the study at any time without giving explanations and without negative effects or repercussions on their medical care.

The present study complies with the ethical principles for medical research on human beings included in the 2013 Declaration of Helsinki.

**BIBLIOGRAFÍA**

-Ackerman IN, Bennell KL. (2004). Austral J Physiother. 50:25–30.

-Alemano F., Houdayer E., Emedoli, D. (2019). PLoS ONE 14(5): e0216858.

-Al Qaraghli, M. I., & De Jesus, O. (2021). Lumbar Disc Herniation.

-Atlas SJ, Keller RB, Chang Y, Deyo RA, Singer DE. (2001). Spine (Phila Pa 1976); 26:1179–1187.

-Asch HL, Lewis PJ, Moreland DB et al. (2002). J Neurosurg.;96:34–44.

-Bagley C, MacAllister M, Dosselman L et al. Current concepts and recent advances in understanding and managing lumbar spine stenosis. F1000Research 2019, 8(F1000 Faculty Rev):137

-Bayraktar, D., Guclu-Gunduz, A., Lambeck, et al. (2016). Disability and Rehabilitation, 38(12), 1163–1171.

-Brox JI, Storheim K, Grotle M, et al. (2008). Spine J. (6):948-958.

-Carli F, Mayo N. (2001). Br J Anaesth;87:531–3.

- Chad, DA. Lumbar spinalstenosis. Neurologic Clinics. 2007;25(2):407-18.

-Choi, G., Raiturker, P. P., Kim, et al., (2005). Neurosurgery, 57(4), 764–772.

- Danielsen, J. M., Johnsen, R., Kibsgaard, S. K., & Hellevik, E. (2000). Spine, 25(8), 1015–1020.

-Demir, S., Dulgeroglu, D., & Cakci, A. (2014). European Journal of Physical and Rehabilitation Medicine, 50(6), 627–640.

- Deyo RA, Mirza SK, Martin BI, et al.: Trends, major medical complications, and charges associated with surgery for lumbar spinal stenosis in older adults. JAMA. 2010; 303(13): 1259– 65.

- Dolan, P., Greenfield, K., Nelson, R. J., & Nelson, I. W. (2000). Spine, 25(12), 1523–1532

-Erdogmus, C. B., Resch, K.-L., Sabitzer, R., et al. (2007). Spine, 32(19), 2041–2049.

- Filiz, M., Cakmak, A., & Ozcan, E. (2005). Clinical Rehabilitation, 19(1), 4–11.

-Fjeld OR, Grøvle L, Helgeland J, et al. (2019). Bone Joint J;101-B:470-7.

-França, F. J., Nogueira Burke, T., Oliveira Magalhães, et al. (2019). American Journal of Physical Medicine & Rehabilitation, 98(3), 207–214.

-Gadjradj PS, Arts MP, Van Tulder MW, et al. (2017). Spine (Phila Pa 1976).

-Gaowgzeh, R. A. M., Chevidikunnan, M. F., BinMulayh, E. A., & Khan, F. (2020). Journal of Back & Musculoskeletal Rehabilitation, 33(2), 225–231.

-Goudman, L., Huysmans, E., Ickmans, et al. (2019). Physical Therapy, 99(7), 933–945.

-Harvie, D. S. et al. (2015). Psychological Science, 26(4), 385–392.

- Kalichman L, Cole R, Kim DH, et al.: Spinal stenosis prevalence and association with symptoms: the Framingham Study. Spine J. 2009; 9(7): 545–50.

-Kamper SJ, Apeldoorn AT, Chiarotto A, Smeets RJ, et al. (2014). Cochrane Database of Systematic Reviews. 9. Art. No.: CD000963.

- Kenney MP, Milling LS. (2016). Theory, Research, and Practice. 3(3):199-210.

- Kim, S.-S., Min, W.-K., Kim, J.-H., & Lee, B.-H. (2014). Journal of Physical Therapy Science, 26(4), 549–552.

-Klinger R, Geiger F, Schiltenwolf M. (2008). Orthopade.37:1002–1006

- Kreiner DS, ShafferWO, Baisden JL, et al. (2013). Spine J.13:734–43.

-Kreiner DS, Hwang SW, Easa JE et al. (2014). Spine J. 14:180–191.

- Lemanu D, Singh P, MacCormick A, et al. (2013). World J Surg. 37:711–20.

-Liu W, Li Q, Li Z, et al. (2019. Medicine (Baltimore);98: e14682.

-Louw A, Diener I, Butler DS, Puentedura EJ. (2011). Archives of physical medicine and rehabilitation.92(12):2041-2056.

- Louw A, Butler DS, Diener I, et al. (2013). Am J Phys Med Rehabil 92:446-52.

-Louw A, Diener I, Landers MR, Puentedura EJ. (2014). Spine (Phila Pa 1976).39:1449–1457

-Louw A, Diener I, Puentedura EJ. (2015). Int J Spine Surg 9:11.

- Lurie JD, Birkmeyer NJ, Weinstein JN. (2003). Spine. 28(6):616-620.

- Macedo LG, Latimer J, Maher CG, et al. (2012). Phys Ther. 92(3):363-377.

-Maier-Riehle B, Harter M. (2001). Int J Rehabil Res.24:199–206.

- Mannion, A. F., Denzler, R., Dvorak, J., et al. (2007). European Spine Journal: Official Publication of the European Spine Society, the European Spinal Deformity Society, and the European Section of the Cervical Spine Research Society, 16(8), 1101–1117.

-Marchand, A. A., O’Shaughnessy, J., Chatillon, C. E., et al. (2016 Journal of Manipulative and Physiological Therapeutics. 39(9), 668-692.

- Matheve, T., Bogaerts, K., & Timmermans, A. (2020). Journal of Neuroengineering and Rehabilitation, 17(1), 55.

-Mbada Ch., Makinde M., Odole A. Et al. (2019). Human Movement. 20(3): 66– 79

-Melzack R. (2001). J Dent Educ.65:1378-82.

-Moseley L. (2002). Aust J Physiother. 48(4):297-302

-Moseley GL. (2003a). Man Ther.8:130-40.

- Moseley GL. (2003b). Journal of Manual & Manipulative Therapy.11(2):88-94.

-Moseley GL. (2004a). Eur J Pain.8(1):39-45.

-Moseley GL, Nicholas MK, Hodges PW. (2004b). Clin J Pain.20(5):324-330.

-Moseley GL. (2005). Aust J Physiother. 51(1):49-52.

- Nambi, G., Abdelbasset, W. K., Elsayed, et al. (2020). Evidence-Based Complementary and Alternative Medicine: ECAM, 2020, 2981273.

-Nielsen PR, Jorgensen LD, Dahl B, et al. (2010). Clin Rehabil. 24:137–48.

-Nygaard OP, Romner B, Trumpy JH. (1994). Acta Neurochir (Wien). 128:53–56.

-Oosterhuis T, Costa LOP, Maher CG, de Vet HCW et al. (2014). Cochrane database Syst Rev.

- Ostelo RW, Costa LO, Maher CG, de vet HC, van Tulder MW. (2009). Spine. 34:1839-48

- Park, J.-H., Lee, S.-H., & Ko, D.-S. (2013). Journal of Physical Therapy Science, 25(8), 985–988.

-Patil A, Chugh A, Gotecha S, Kotecha M, (2018). J Craniovert Jun Spine; 9:156-62.

-Paulsen, R. T., Rasmussen, J., Carreon, L. Y., & Andersen, M. (2020). The Spine Journal: Official Journal of the North American Spine Society, 20(1), 41–47.

- Rathod TN, Chandanwale A, Ladkat KM, et al. (2014). Indian J Orthop. 48:354–359.

- Reiter, K. (2014). AORN Journal, 99(3), 376-384.

- Saal JA, Saal JS. (1991). Philadelphia: Lea and Febiger; 29: 318–327.

- Santa Mina D, Clarke H, Ritvo P, et al. (2014). Physiotherapy.100:196–207.

-Shen, Y. J., & Huang, S. Z. (2005). Medicare Studies, 1, 85-93.

-Suri P., Pearson A., Scherer E. (2016). PM R. ; 8(5): 405–414.

- Taylor RS, Taylor RJ. (2012). Br J Pain. 6:174–181

- Tomkins-Lane CC, Battié MC, Hu R, et al.: Pathoanatomical characteristics of clinical lumbar pinal stenosis. J Back Musculoskelet Rehabil. 2014; 27(2): 223–9.

- Tomkins-Lane CC, Lafave LM, Parnell JA, Rempel J, Moriartey S, Andreas Y, et al. The spinal stenosis pedometer and nutrition lifestyle intervention (SSPANLI): development and pilot. The

Spine Journal 2015;15(4):577-86.

- Valkenet K, van de Port IG, Dronkers JJ, et al. (2011). Clin Rehabil.25:99–111.

-Voorhies RM, Jiang X, Thomas N. (2007). Spine J. 7:516–524.

-Waddell G.(2004). 2nd ed. (Livingstone C, ed.). Churchill Livingstone

-Wang, R. Q., Lin, Y. H., & Chen, Z. Y. (2016). Physical Therapy, 41(2), 166-167.

-Wang, J., Chen, L., Yu, M., & He, J. (2020). Annals of Palliative Medicine, 9(2), 388–393.

-Watters WC, Bono CM, Gilbert TJ, et al. (2009). Spine J.609–14.

-Watters WC, Baisden J, Gilbert TJ, Kreiner S, Resnick DK,Bono CM, et al. Degenerative lumbar spinal stenosis: an evidence-based clinical guideline for the diagnosis and treatment of degenerative lumbar spinal stenosis. The Spine Journal 2008;8(2):305-10.

-Weinstein JN, Lurie JD, Tosteson TD, et al. (2006). JAMA. 296(20):2451-2459.

- Yílmaz, F., Yílmaz, A., Merdol, F., et al. (2003). Journal of Rehabilitation Medicine, 35(4), 163–167.

- Yilmaz Yelvar, G. D., Çırak, Y., Dalkılınç, M., et al. (2017European Spine Journal : Official Publication of the European Spine Society, the European Spinal Deformity Society, and the European Section of the Cervical Spine Research Society, 26(2), 538–545.

-Yoo, J.-H., Kim, S.-E., Lee, M.-G., et al. (2014). International Journal of Clinical Practice, 68(8), 941–949.

- Zaina F, Tomkins-Lane C, Carragee E, Negrini S. Surgical versus non-surgical treatment for lumbar spinal stenosis. Cochrane Database of Systematic Reviews 2016, Issue 1. Art. No.: CD010264.
